# GPX2+ tumor cells recruit LGALS1+ B cells via CCL26-CCR3 axis to promote immunosuppression and tumor progression in hepatocellular carcinoma

**DOI:** 10.3389/fimmu.2026.1709855

**Published:** 2026-03-20

**Authors:** Liang Lin, Shiye Yang, Jixiang Zhang, Guodu Chen, Wuhan Zhou, Dongxing Chen, Jiafei Chen

**Affiliations:** 1 Department of Hepatobiliary Surgery, The First Hospital of Putian City, Putian, Fujian, China; 2 Department of Comprehensive Surgery, Nantong First People’s Hospital, Affiliated Hospital 2 of Nantong University, Nantong, Jiangsu, China; 3 General Surgery Department I, Zhongshan City People’s Hospital, No. 2, Zhongshan, Guangdong, China

**Keywords:** B cell, CSCs, GPX2, HBV-positive HCC, ROS

## Abstract

The molecular link between Hepatitis B virus (HBV) infection and hepatocellular carcinoma (HCC) progression remains elusive. Here, we identify glutathione peroxidase 2 (GPX2) as a pivotal mediator of this process. Single-cell analysis of HBV-positive HCC reveals a distinct GPX2^+^ CSC population characterized by high MYC and CD44 expression. We demonstrate that GPX2 preserves stemness intrinsically by mitigating ROS-mediated c-MYC nuclear-cytoplasmic distribution, while extrinsically fostering immune evasion via the CCL26-CCR3 signaling axis. specifically, GPX2-derived CCL26 recruits and educates B cells towards an immunosuppressive LGALS1^+^ state, which predicts adverse patient outcomes. *In vivo*, GPX2 overexpression accelerates tumorigenesis, whereas targeting CCR3 with ALK4290 sensitizes tumors to anti-PD-1 checkpoint blockade. These findings delineate a dual mechanism whereby GPX2 couples oxidative stress regulation to immune modulation, positioning the GPX2-B cell axis as a promising therapeutic target for HBV-driven liver cancer.

## 1 Introduction

Hepatitis B virus (HBV) infection represents a global health crisis and is the principal etiological driver of hepatocellular carcinoma (HCC), the most prevalent form of primary liver cancer (1). Epidemiological studies have firmly established that chronic HBV infection elevates the risk of developing HCC by 10- to 25-fold, often following a pathological progression through chronic hepatitis and cirrhosis (2, 3). Despite this unequivocal link, the precise molecular mechanisms by which HBV orchestrates malignant transformation and fosters an environment conducive to tumor growth remain incompletely understood. This knowledge gap is particularly critical concerning the processes that distinguish HBV-driven hepatocarcinogenesis from that arising from other etiologies, such as alcohol consumption or metabolic syndrome.

A central hallmark of HBV-associated HCC is the dysregulation of cellular programs that govern self-renewal and differentiation, leading to the emergence and maintenance of a subpopulation of cancer stem-like cells (CSCs) (4, 5). These CSCs are considered the primary drivers of tumor initiation, therapeutic resistance, and metastatic relapse. While previous research has implicated HBV in the modulation of canonical stemness pathways, including Wnt, MAPK, and Notch, the specific effectors that functionally connect viral presence to the CSC phenotype are not fully elucidated (6–9). Concurrently, the tumor microenvironment (TME), characterized by a complex interplay of redox homeostasis and immune cell activity, provides critical extrinsic cues that sustain the CSC niche (10, 11). A key regulator of this environment is the glutathione peroxidase (GPX) family, whose members, such as GPX2, are crucial for managing intracellular reactive oxygen species (ROS) (12). Although GPX2 is recognized as an oncogene in several malignancies, its specific contribution to the CSC state in HCC remains a significant and unexplored area of inquiry (13, 14).

Furthermore, the influence of CSCs extends beyond intrinsic tumor cell biology; they actively remodel the TME to facilitate immune evasion (15). CSCs are known to secrete a host of immunosuppressive factors and can subvert anti-tumor immunity by interfering with the function of effector immune cells like dendritic cells and T cells (16). However, while the general paradigm of CSC-immune interplay is established, the specific mechanisms by which CSCs sculpt the immune landscape in the distinct context of HBV-positive HCC are poorly characterized. This leaves a critical gap in our understanding of how HBV-associated tumors evade immune surveillance and progress.

In this study, we leveraged single-cell transcriptomic analysis of human HCC tissues to address these fundamental questions. We first identified a significant enrichment of a GPX2-expressing (GPX2^+^) tumor cell population in HBV-positive HCC, which exhibits robust CSC characteristics. We proceeded to elucidate a novel mechanism wherein GPX2 promotes and sustains the CSC phenotype by suppressing intracellular ROS, consequently activating the MYC signaling axis. Furthermore, we uncovered a distinct mechanism of immune evasion orchestrated by these GPX2^+^ CSCs. We demonstrate that GPX2 drives the secretion of the cytokine CCL26, which in turn induces an immunosuppressive LGALS1^+^ phenotype in B cells, thereby fostering a tumor-permissive immune microenvironment. Collectively, our findings reveal a dual, interconnected mechanism through which GPX2 not only enhances the intrinsic stem-like properties of HBV-associated HCC but also actively shapes the immune TME to promote tumor progression.

## 2 Materials and methods

### 2.1 Human specimen datasets and single-cell sequencing data processing

RNA sequencing raw counts and corresponding clinical annotations from 371 patients with hepatocellular carcinoma (HCC) were retrieved from The Cancer Genome Atlas (TCGA; https://portal.gdc.cancer.gov/), with sample collection and usage strictly adhering to established guidelines and policies. Correlation analyses were conducted using the cBioPortal platform. For single-cell analyses, we employed the GSE282701 dataset, comprising scRNA-seq profiles from three HBV-negative HCC samples, three HBV-positive HCC samples, and matched normal liver tissues. FASTQ files were processed with the 10x Genomics Cell Ranger pipeline (v6.0.0) using the human reference genome GRCh38-3.0.0 under default parameters. Downstream analyses were performed with the Seurat R package (v4.3.0). Differentially expressed genes were identified using the FindAllMarkers function, while functional enrichment was assessed with the clusterProfiler package under default settings. Feature scoring was conducted with the AddModuleScore function.

### 2.2 HPA and tissue protein level detection

Immunohistochemistry (IHC) images of GPX2 in HCC tissues were retrieved from the Human Protein Atlas (HPA; http://www.proteinatlas.org/). In addition, clinical specimens of HBV-negative and HBV-positive HCC were collected for IHC staining, with all patients providing informed consent prior to sample acquisition. Among the 158 available patient records, 85 were HBV-positive HCC patients and 73 were HBV-negative HCC patients, of whom 44 had a tumor diameter exceeding 7 cm. Primary antibodies included anti-GPX2 (ab137431, Abcam,1:1000), anti-CD44 (ab243894, Abcam,1:250), and anti-ALDH1A1 (ab52492, Abcam,1:500), applied at working concentrations recommended by the manufacturers.

For IHC, paraffin-embedded tissue sections were first baked at 60 °C for over 2 hours, followed by sequential incubation in xylene (10 min) and graded ethanol solutions (5 min each step), before rinsing in distilled water. Antigen retrieval was performed by microwave heating, after which slides were cooled to room temperature and blocked with bovine serum albumin (BSA). Sections were then incubated with primary antibodies overnight at 4 °C. After returning to room temperature, slides were treated with appropriate secondary antibodies, visualized using 3,3′-diaminobenzidine (DAB), and subsequently dehydrated and mounted for analysis.

### 2.3 H-score assessment

The H-score system was applied to quantitatively evaluate immunohistochemistry (IHC) staining by integrating both the proportion of positively stained cells and staining intensity. The score was calculated as:


$$\text{H}-\text{score}=\sum \left({\text{P}}_{i}\times \text{I}\right)$$

where P_i_ denotes the percentage of positive cells at a given staining intensity, and I represents the staining intensity level (1 = weak, 2 = moderate, 3 = strong). The final H-score ranges from 0 to 300, with higher values indicating stronger overall staining.

### 2.4 Immunofluorescence staining

For immunofluorescence staining, HCC cells subjected to different treatments were collected, digested into single-cell suspensions, and seeded onto culture plates. Upon reaching approximately 80% confluence, cells were fixed with 4% paraformaldehyde at room temperature for 20 min, followed by three washes with PBS. Non-specific antigens were blocked using immunofluorescence blocking solution (Beyotime, China) for 30 min. Cells were then incubated with primary antibodies (anti–c-MYC,1:200) overnight at 4 °C. After three additional washes, cells were incubated with the corresponding fluorescently labeled secondary antibodies for 1 h, counterstained with DAPI, and imaged using a fluorescence microscope.

### 2.5 Cell culture

MHCC97H, Hep3B, and JM1 cell lines were obtained from the National Collection of Authenticated Cell Cultures, Chinese Academy of Sciences. MHCC97H cells were maintained in Dulbecco’s Modified Eagle Medium (DMEM; Gibco, C11995500BT) supplemented with 10% fetal bovine serum (FBS), Hep3B cells in Minimum Essential Medium (MEM; Gibco, PM150410) supplemented with 10% FBS, and JM1 cells in a specialized medium (Procell, CM-0987). All media were further supplemented with 1% penicillin–streptomycin (Gibco, 15140-122). Cells were cultured at 37 °C in a humidified incubator (Thermo Fisher Scientific, USA) with 5% CO_2_.

### 2.6 Cell transfection

Plasmid DNA or siRNA was transfected into HCC cell lines using lipid-based transfection method. Cells were cultured in DMEM high glucose medium supplemented with 10% fetal bovine serum and 1% penicillin-streptomycin at 37 °C with 5% CO_2_. Twenty-four hours before transfection, cells were seeded in 24-well plates at a density of 5×10^4^ cells per well to reach 70-80% confluence at the time of transfection. For transfection complex preparation, 1 μg plasmid DNA or 50 nM siRNA was diluted in 50 μL Opti-MEM medium (tube A), while 1.5 μL Lipofectamine 3000 reagent was diluted in 50 μL Opti-MEM (tube B). After 5 minutes of incubation, the solutions from both tubes were combined and incubated at room temperature for 15 minutes. The original cell culture medium was then replaced with 450 μL fresh antibiotic-free medium, followed by dropwise addition of 100 μL transfection complexes. After 4–6 hours of transfection, the medium was replaced with fresh complete medium. Transfection efficiency was evaluated 48 hours post-transfection by Western Blotting or qPCR analysis, while fluorescent plasmids were assessed using fluorescence microscopy. The overexpression plasmid and the shRNA used in the study were both custom-constructed and provided by GenePharma (Shanghai, China). The specific shRNA sequences targeting GPX2 are as follows:

sequence1:

Sense: 5’-GAAGGUAGAUUUCAAUACGUU-3’
Antisense: 5’-AACGUAUUGAAAUCUACCUUC-3’

sequence2:

Sense: 5’-CCCUCUGGUUGGUGAUUCA-3’
Antisense: 5’-UGAAUCACCAACCAGAGGG-3’

### 2.7 CCK8 cell proliferation assay

Cell proliferation was assessed using the Cell Counting Kit-8 (CCK-8; Beyotime, China) according to the manufacturer’s instructions. Briefly, MHCC97H and Hep3B cells with GPX2 overexpression or knockdown were resuspended and seeded into 96-well plates. After 48 hours of incubation, the culture medium was replaced with 100 μl of fresh medium containing 10 μl of CCK-8 solution. Cells were further incubated for 2 hours at 37 °C in the dark, and absorbance was measured at 450 nm using a microplate reader (BioTek, Winooski, VT, USA).

### 2.8 ROS analyses

Intracellular ROS levels were assessed by flow cytometry. When cell confluence reached approximately 80%, cells were harvested and washed twice with pre-chilled PBS, then resuspended in 1× binding buffer. A 100 μl aliquot of the suspension was incubated with DCFH-DA (1:1000; Beyotime, China) for 15 min at 37 °C in the dark. The reaction was terminated by adding 400 μl of 1× binding buffer. Samples were analyzed within 1 h on a BD FACSCanto II flow cytometer (BD Biosciences, USA), with excitation at 488 nm and fluorescence detection in the FITC channel (530/30 nm). Forward and side scatter (FSC/SSC) gating was applied, and single-stained controls were used to adjust compensation. A total of 10,000 events were collected in logarithmic mode. Data were processed and analyzed using FlowJo software (v10.0).

### 2.9 Western blot

Western blotting was performed to assess protein expression in HCC cells following treatment with various concentrations of extracts. Cells were lysed in ice-cold RIPA buffer (Beyotime, P0013B) supplemented with PMSF (Beyotime, ST506). Total protein concentrations were determined using a NanoDrop 2000/2000C spectrophotometer (Thermo Fisher Scientific, USA). Equal amounts of protein were resolved by SDS–PAGE and transferred onto PVDF membranes (Millipore, IPVH00010). Membranes were blocked with 5% non-fat milk and incubated with primary antibodies, followed by incubation with the corresponding secondary antibodies after three washes in TBST. Protein bands were visualized using a chemiluminescence imaging system (Tanon, China). The antibodies were used as GPX2(Santa cruz, sc-133160,1:50), GAPDH(Proteintech, 1E6D9, 1:20000).

### 2.10 ELISA

The concentration of CCL26 in spinal cord tissue homogenates was quantified using a commercial ELISA kit (R&D Systems, Catalog # DCC260B) according to the manufacturer’s protocol. Briefly, tissue supernatants were obtained through homogenization and centrifugation, with total protein concentration determined by BCA assay. Samples were appropriately diluted to fall within the assay’s linear range (15.6–1000 pg/mL) and analyzed in duplicate. The assay procedure included incubation with samples and standards, washing steps, and sequential incubation with biotinylated detection antibody and streptavidin-HRP conjugate. After TMB substrate development and reaction termination, absorbance was measured at 450 nm with wavelength correction. CCL26 concentrations were determined from the standard curve using four-parameter logistic regression and normalized to total protein content (pg/mg protein).

### 2.11 Statistical analyses

Statistical analyses were performed as following statement: For two-group comparisons, unpaired Student’s t-test was used. For comparisons involving three or more groups, one-way ANOVA was applied. Tumor growth curves were analyzed using two-way repeated-measures ANOVA. A P value of less than 0.05 was considered statistically significant, with significance levels denoted as follows: P < 0.05 (*), P < 0.01 (**), and P < 0.001 (***).

## 3 Results

### 3.1 Profiling and characterization of hepatocellular carcinoma at single-cell resolution

**Figure 1:**
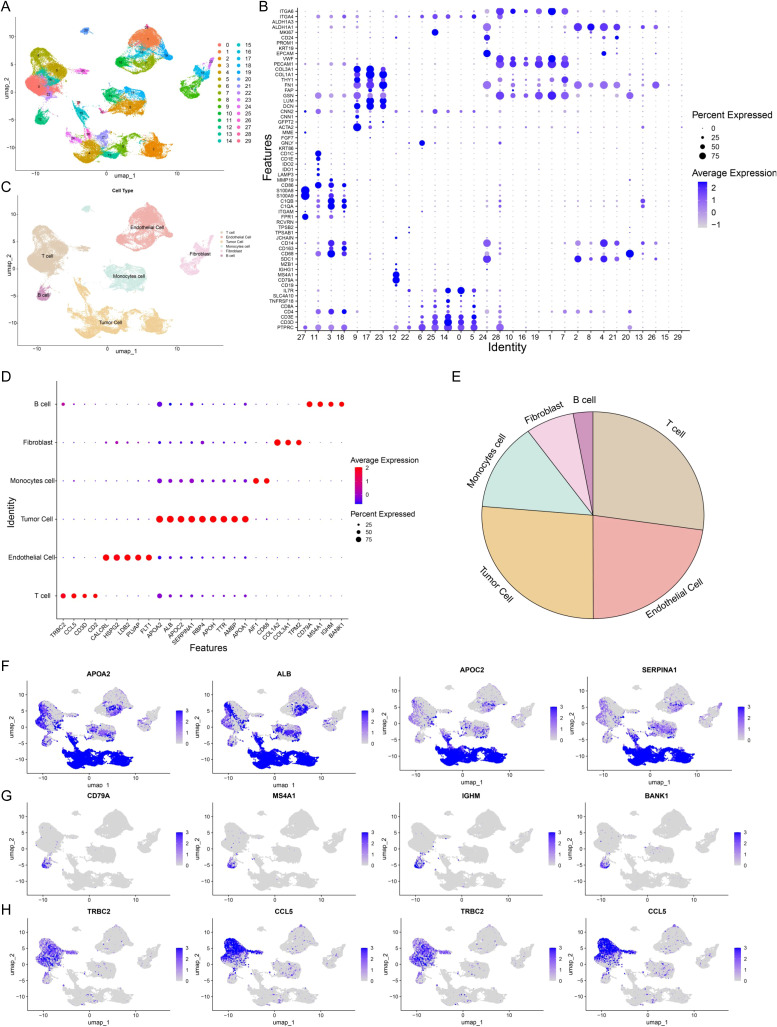
Profiling and characterization of hepatocellular carcinoma at single-cell resolution. **(A)** UMAP visualization of scRNA-seq data from all HCC samples, color-coded by clusters. Each cluster was annotated according to cluster-specific marker genes shown in **(B)**. **(B)** Dot plot displaying representative marker genes for each major cluster. Color scale indicates the average normalized expression, while dot size reflects the proportion of cells expressing the corresponding marker within each cluster. **(C)** UMAP visualization of scRNA-seq data from all HCC samples, color-coded by cell type. Each cell type was annotated based on marker genes shown in **(D)**. **(D)** Dot plot of representative marker genes for each major cell type. Color scale indicates average normalized expression, and dot size corresponds to the fraction of marker-positive cells within each cell type. **(E)** Pie chart showing the relative proportions of each cell type. **(F)** Feature plots of representative tumor cell markers (APOA2, ALB, APOC2, SERPINA1). **(G)** Feature plots of representative B-cell markers (CD79A, MS4A1, IGHM, BANK1). **(H)** Feature plots of representative T-cell markers (CD3D, CD2, TRBC2, CCL5).

The large-scale integration of single-cell RNA sequencing (scRNA-seq) data from a multitude of patients has enabled the construction of a high-resolution atlas of the hepatocellular carcinoma (HCC) landscape. To conduct a comparative analysis based on hepatitis B virus (HBV) infection status, we curated a composite single-cell transcriptomic dataset from the GSE282701 dataset. This dataset comprised 12 liver-associated samples, including six normal liver tissues and six tumor tissues. The latter were further stratified into HBV-negative (n=3) and HBV-positive (n=3) HCC specimens. Following rigorous quality control and the removal of batch effects (see Materials and Methods), the scRNA-seq profiles of the remaining cells were retained for downstream analysis. Accordingly, the integrated dataset was partitioned into two principal cohorts: an HBV-negative group and an HBV-positive group. Through unsupervised clustering and subsequent cluster identification, we initially resolved the entire cell population into 29 distinct clusters (**Figures 1A, B**). These clusters were then annotated based on canonical marker genes and consolidated into six major cell types: T cells, endothelial cells, malignant cells, monocytes, fibroblasts, and B cells (**Figure 1C**).

**Figure 1D** highlights the canonical marker genes that define the distinct cell populations (**Figure 1E**). The T-cell cluster was characterized by the expression of TRBC2, CCL5, CD3D, and CD2, whereas endothelial cells were identified by markers including CALCRL, HSPG2, LDB2, and PLVAP. Malignant cells exhibited prominent expression of APOA2, ALB, APOC2, SERPINA1, RBP4, APOH, TTR, AMBP, and APOA1 (**Figures 1F–H**). Furthermore, monocytes were distinguished by AIF1 and CD68; fibroblasts by COL1A2, COL1A3, and TPM2; and B cells by CD79A, MS4A1, IGHM, and BANK1 (**Supplementary Figures 1B, C**). A corresponding pie chart illustrates the proportional abundance of each cell population within the entire dataset. Subsequently, we stratified the entire sample cohort into HBV-negative and HBV-positive groups to investigate the differential abundance of the constituent cell populations between these two conditions.

### 3.2 Characterization and functional profiling of distinct tumor cell populations in HBV-negative and HBV-positive hepatocellular carcinoma

**Figure 2:**
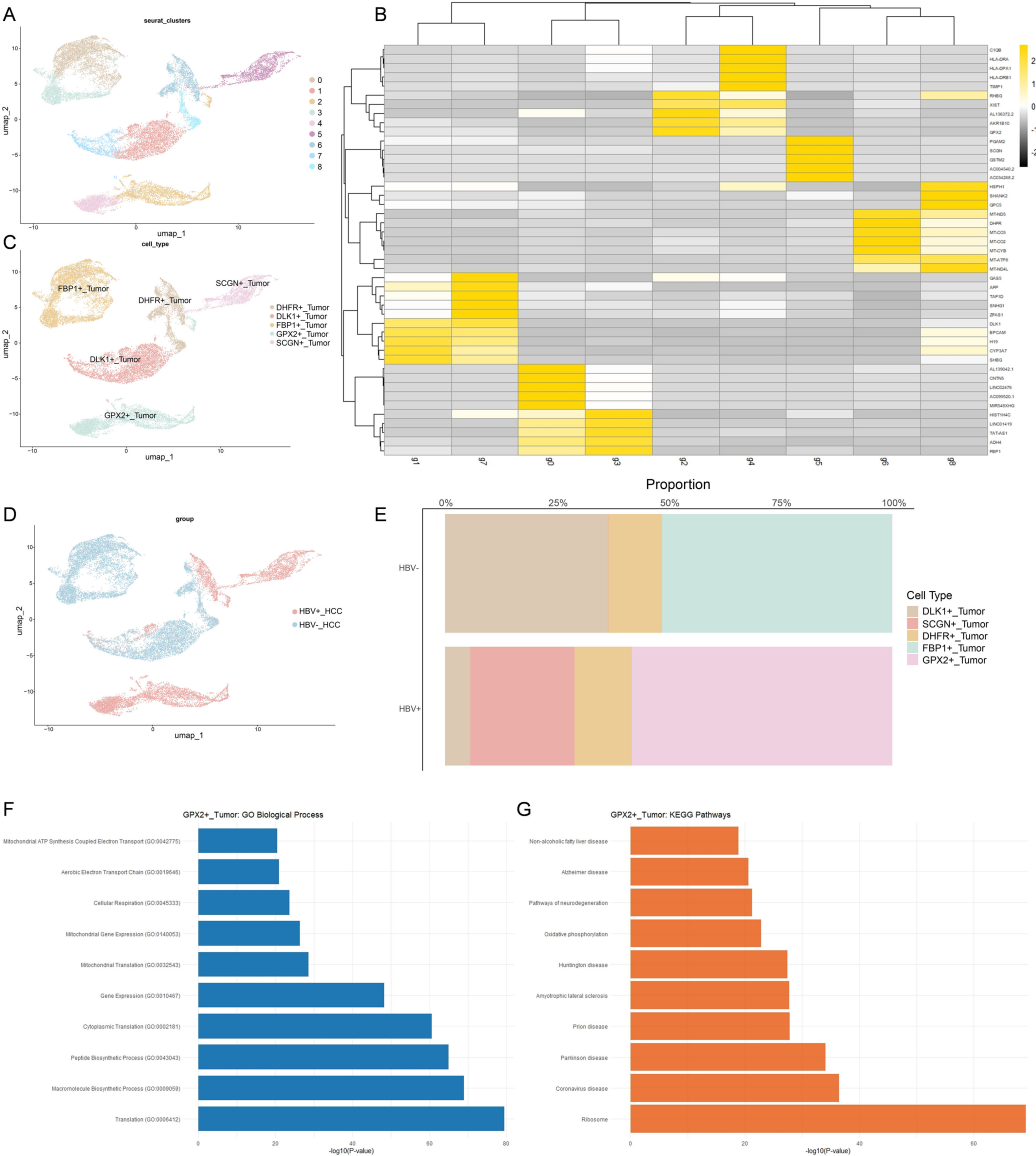
Characterization and functional profiling of distinct tumor cell populations in HBV-negative and HBV-positive hepatocellular carcinoma. **(A)** UMAP visualization of scRNA-seq data from tumor cells across all HCC samples, color-coded by clusters. Clusters were annotated according to cluster-specific marker genes shown in **(B)**. **(B)** Heatmap of representative marker genes for each major cluster. **(C)** UMAP visualization of tumor cells from all HCC samples, color-coded by subpopulation identity. **(D)** Distribution of distinct tumor cell subpopulations in HBV-negative and HBV-positive HCC. **(E)** Bar plot showing the relative abundance of tumor cell subpopulations in HBV-negative versus HBV-positive HCC. **(F)** GO enrichment analysis of characteristic genes from GPX2^+^ tumor cell subpopulations. **(G)** KEGG enrichment analysis of characteristic genes from GPX2^+^ tumor cell subpopulations.

To delineate the cellular heterogeneity associated with HBV status in hepatocellular carcinoma (HCC), we next examined tumor cells in greater detail, as they represent the principal population of interest. Following dimensionality reduction, tumor cells were partitioned into nine distinct subclusters, each defined by unique marker gene expression profiles (**Figures 2A, B**). These subclusters were further refined and re-annotated into five major categories on the basis of their characteristic genes, yielding DHFR^+^ tumor cells, DLK1^+^ tumor cells, FBP1^+^ tumor cells, GPX2^+^ tumor cells, and SCGN^+^ tumor cells (**Figure 2C**). This stratification highlights the diverse transcriptional programs operating within malignant cells and provides a framework for dissecting functional differences between HBV-negative and HBV-positive HCC.

Subsequently, we projected tumor cells onto UMAP embeddings stratified by patient groups to visualize the distribution of cellular populations under different HBV infection statuses. Bar plot analyses further quantified the proportional composition of each cell type across HBV-negative and HBV-positive HCC (**Figure 2D**). Strikingly, this comparison revealed the presence of a distinct subset of tumor cells characterized by high GPX2 expression, which was predominantly enriched in HBV-positive patients (**Figure 2E**). To investigate the functional characteristics of the stratified tumor cell subpopulations, we performed Gene Ontology (GO) and Kyoto Encyclopedia of Genes and Genomes (KEGG) enrichment analyses using the highly expressed genes of each group. KEGG enrichment revealed that GPX2^+^ tumor cells were significantly enriched in the oxidative phosphorylation and ribosome pathways (**Figure 2G**). Consistently, GO enrichment highlighted a strong association of GPX2^+^ tumor cells with mitochondrial-related processes, including mitochondrial gene expression and mitochondrial translation (**Figure 2F**). These findings suggest that GPX2^+^ tumor cells may possess distinct metabolic and translational programs, particularly linked to mitochondrial activity. In addition to GPX2^+^ tumor cells, GO and KEGG enrichment analyses of the remaining four tumor cell populations revealed distinct functional signatures unique to each group. These findings underscore the functional heterogeneity among tumor subpopulations and highlight specialized biological programs operating within different cellular compartments of HCC.

### 3.3 GPX2 drives the expression of stemness-associated features in hepatocellular carcinoma cells

**Figure 3:**
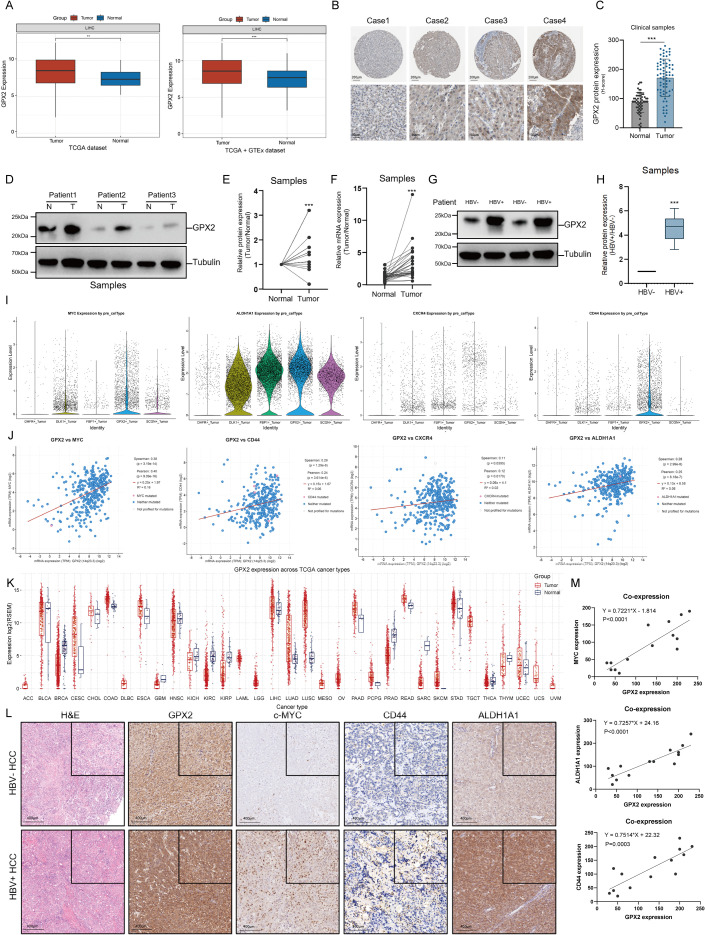
GPX2 drives the expression of stemness-associated features in hepatocellular carcinoma cells. **(A)** mRNA and protein expression of GPX2 in tumor and normal tissues from the TCGA and GTEx datasets. **(B)** Representative IHC images showing differential GPX2 expression from the Human Protein Atlas (HPA). **(C)** Quantification of GPX2 protein expression by IHC in cour cohort. **(D)** GPX2 protein levels in surgically resected clinical samples, with Tubulin as the loading control. **(E)** Quantification of GPX2 protein expression. **(F)** GPX2 mRNA levels in clinical HCC samples. **(G)** GPX2 protein expression in HBV-negative versus HBV-positive HCC tissues. **(H)** GPX2 mRNA expression in HBV-negative versus HBV-positive HCC tissues. **(I)** Expression of stemness-associated markers (MYC, ALDH1A1, CXCR4, CD44) across tumor cell subtypes at the single-cell level. **(J)** Correlation analysis between GPX2 and stemness-related markers (MYC, CD44, CXCR4, ALDH1A1) in the TCGA-LIHC cohort. **(K)** GPX2 expression across multiple tumor types in the TCGA dataset. **(L)** Representative H&E and IHC staining of GPX2, c-MYC, CD44, and ALDH1A1 in clinical samples. **(M)** Correlation analysis of GPX2 with MYC, ALDH1A1, and CD44 expression in clinical HCC tissues. *** P<0.001.

Given that our study primarily focused on key features of HBV-positive hepatocellular carcinoma (HCC), we selected GPX2^+^ tumor cells as the main population for further investigation. We first examined GPX2 expression in the TCGA-LIHC and GTEx datasets, comparing tumor tissues with normal liver tissues at both the mRNAxand protein levels. The results consistently demonstrated that GPX2 expression was elevated in tumor tissues relative to normal counterparts (**Figure 3A**). Moreover, analysis of immunohistochemistry (IHC) data from the Human Protein Atlas (THPA) further revealed heterogeneous GPX2 expression across different HCC samples (**Figure 3B**). Beyond the THPA findings, tissue microarray samples derived from HBV-positive and HBV-negative hepatocellular carcinoma patients were subjected to GPX2 immunohistochemical staining and quantitative analysis. The results aligned with data from public databases, further validating the association between GPX2 expression and HBV infection status in clinical specimens (**Figure 3C**; **Supplementary Figures 4A, B**). To validate these observations, we collected clinical HCC samples along with adjacent normal liver tissues from different patients, stratified into two groups according to HBV infection status. Proteins and RNAs were extracted from both tumor and matched normal tissues and analyzed by Western blotting and quantitative PCR (qPCR). Consistent with the TCGA-based analyses, GPX2 expression was markedly elevated in tumor tissues relative to adjacent normal counterparts (**Figures 3D–F**). Furthermore, subgroup analyses revealed that GPX2 levels were significantly higher in HBV-positive HCC tissues compared with HBV-negative cases, as confirmed by both Western blotting and qPCR (**Figures 3G, H**).

Previous studies have reported that HBV-positive hepatocellular carcinoma (HCC) exhibits stronger cancer stem cell–like properties compared with HBV-negative HCC (17, 18). In addition, GPX2 has been shown in non–small cell lung cancer to markedly promote cancer stemness and therapeutic resistance through activation of the Hedgehog signaling pathway (14). Based on these findings, we hypothesized that the GPX2^+^ tumor cell population in HBV-positive HCC may represent a subpopulation with stem cell–like characteristics, and we therefore pursued further investigation. To determine whether GPX2^+^ tumor cells exhibit cancer stem cell–like properties, we examined the expression of established liver cancer stem cell markers, including MYC, CXCR4, CD44, and ALDH1A1, across tumor cell subpopulations in the single-cell dataset (19–22). Notably, these markers were significantly upregulated in the GPX2^+^ tumor cell population compared with other subgroups (**Figure 3I**). This observation suggests that among the five annotated tumor cell clusters, GPX2^+^ cells possess the most pronounced stemness features. We next assessed the correlation between GPX2 and liver cancer stem cell markers (MYC, CXCR4, CD44, and ALDH1A1) in the TCGA-LIHC dataset. Consistent with the single-cell analysis, bulk RNA sequencing data demonstrated a positive correlation between GPX2 and these stemness-associated markers (**Figure 3J**). Moreover, a pan-cancer analysis revealed that GPX2 expression was relatively elevated in LIHC compared with other tumor types (**Figure 3K**). The corresponding pan-cancer profiles of MYC, CXCR4, CD44, and ALDH1A1 are provided in the supplementary figures (**Supplementary Figure 2**).

We further validated these findings in clinical HCC specimens. Consecutive sections from HBV-negative and HBV-positive tumors were subjected to hematoxylin and eosin (H&E) staining, as well as immunohistochemical (IHC) staining for GPX2, c-MYC, CD44, and ALDH1A1(**Figure 3L**). Consistent with the transcriptomic analyses, GPX2 staining was markedly stronger in HBV-positive samples compared with HBV-negative tumors. Notably, elevated expression of c-MYC, CD44, and ALDH1A1 was also observed in HBV-positive tissues. Quantitative assessment using H-scores, followed by correlation analyses, confirmed that GPX2 expression levels were positively associated with c-MYC, CD44, and ALDH1A1 in HCC tissues, in agreement with the TCGA dataset(**Figure 3M**). Collectively, these findings indicate that in HBV-positive hepatocellular carcinoma, GPX2 may function as a cancer stem cell–associated molecule, playing a pivotal role in promoting stemness-related properties of tumor cells.

### 3.4 GPX2 promotes malignant phenotypes and cancer stem cell properties in HBV-positive hepatocellular carcinoma

**Figure 4:**
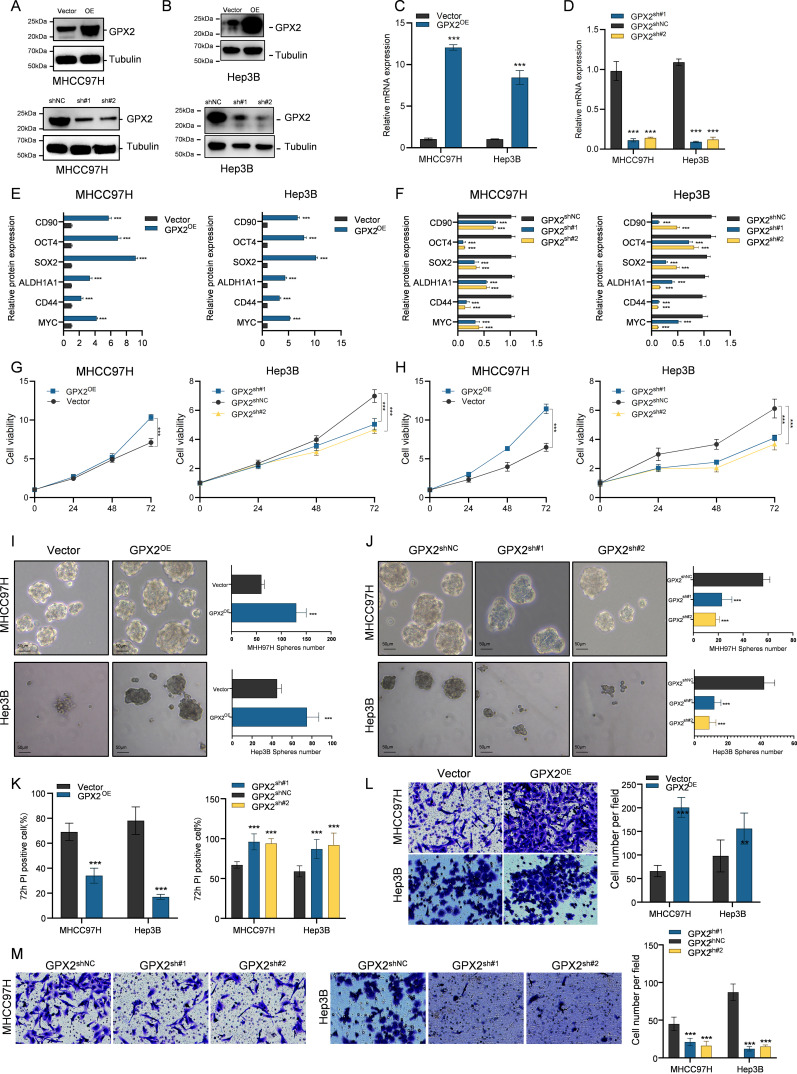
GPX2 promotes malignant phenotypes and cancer stem cell properties in HBV-positive hepatocellular carcinoma. **(A, B)** Western blot analysis confirming GPX2 overexpression and knockdown efficiency in MHCC97H **(A)** and Hep3B **(B)** cells. **(C)** qPCR validation of GPX2 overexpression efficiency at the mRNA level in MHCC97H and Hep3B cells. **(D)** qPCR validation of GPX2 knockdown efficiency at the mRNA level in MHCC97H and Hep3B cells. **(E)** Alterations in stemness-related gene expression following GPX2 overexpression in MHCC97H and Hep3B cells. **(F)** Alterations in stemness-related gene expression following GPX2 knockdown in MHCC97H and Hep3B cells. **(G)** Cell proliferation rates in MHCC97H and Hep3B cells after GPX2 overexpression. **(H)** Cell proliferation rates in MHCC97H and Hep3B cells after GPX2 knockdown. **(I, J)** Sphere formation capacity and quantification in MHCC97H and Hep3B cells upon GPX2 overexpression **(I)** or knockdown **(J)**. **(K)** PI staining assay assessing cisplatin sensitivity in GPX2-overexpressing or knockdown cells. **(L, M)** Migration capacity and quantification in MHCC97H and Hep3B cells following GPX2 overexpression **(L)** or knockdown **(M)**. *** P<0.001.

We next investigated the functional role of GPX2 in HBV-positive hepatocellular carcinoma (HCC) cell lines. Two representative HBV-positive HCC cell lines, MHCC97H and Hep3B, were selected, and GPX2 expression was manipulated by plasmid-mediated overexpression and shRNA-mediated knockdown. The efficiency of these manipulations was confirmed by Western blotting and qPCR (**Figures 4A–D**). As expected, GPX2 protein and mRNA levels were robustly increased in overexpression cells, whereas they were markedly reduced in knockdown cells, compared with empty vector or control groups. We next assessed the impact of GPX2 on the expression of liver cancer stem cell–associated markers in MHCC97H and Hep3B cells. RNA was extracted from GPX2-overexpressing and GPX2-knockdown cells, and the expression levels of representative stemness markers were quantified. In both cell lines, GPX2 overexpression markedly upregulated CD90, OCT4, SOX2, ALDH1A1, CD44, and MYC, whereas GPX2 knockdown led to a significant reduction in the expression of these markers (**Figures 4E, F**). These results indicate that GPX2 positively regulates the stemness-associated transcriptional program in HBV-positive HCC cells.

We further examined the functional impact of GPX2 on HBV-positive HCC cells. CCK-8 assays were performed in MHCC97H and Hep3B cells to assess proliferative capacity following GPX2 overexpression or knockdown. The results showed that GPX2 overexpression significantly accelerated cell proliferation, whereas GPX2 silencing markedly suppressed growth (**Figures 4G, H**). To evaluate stemness-related properties, we next conducted sphere formation assays, a well-established method for assessing cancer stem cell potential. GPX2-overexpressing or GPX2-knockdown cells were seeded at a density of 800 cells per well in low-adhesion culture plates and maintained for 72 hours. Quantification of tumor spheres revealed that GPX2 overexpression substantially enhanced sphere-forming capacity in both cell lines, whereas GPX2 knockdown significantly impaired sphere formation (**Figures 4I, J**). Chemoresistance is another hallmark feature associated with cancer stem cell properties. To assess this, MHCC97H and Hep3B cells were treated with cisplatin for 72 hours, followed by propidium iodide (PI) staining to evaluate apoptosis. The results showed that GPX2 overexpression markedly enhanced resistance to cisplatin-induced apoptosis, whereas GPX2 knockdown sensitized tumor cells to chemotherapy (**Figure 4K**). In addition, Transwell migration assays were performed to examine the effects of GPX2 on cell motility. GPX2 overexpression significantly promoted the migratory capacity of HCC cells, while GPX2 silencing led to a pronounced reduction in migration (**Figures 4L, M**).

In summary, these experimental findings demonstrate that GPX2 significantly promotes malignant phenotypes in hepatocellular carcinoma cells, with a particularly profound impact on cancer stem cell–associated properties.

### 3.5 GPX2 promotes hepatocellular carcinoma stemness via the ROS–MYC signaling axis

**Figure 5:**
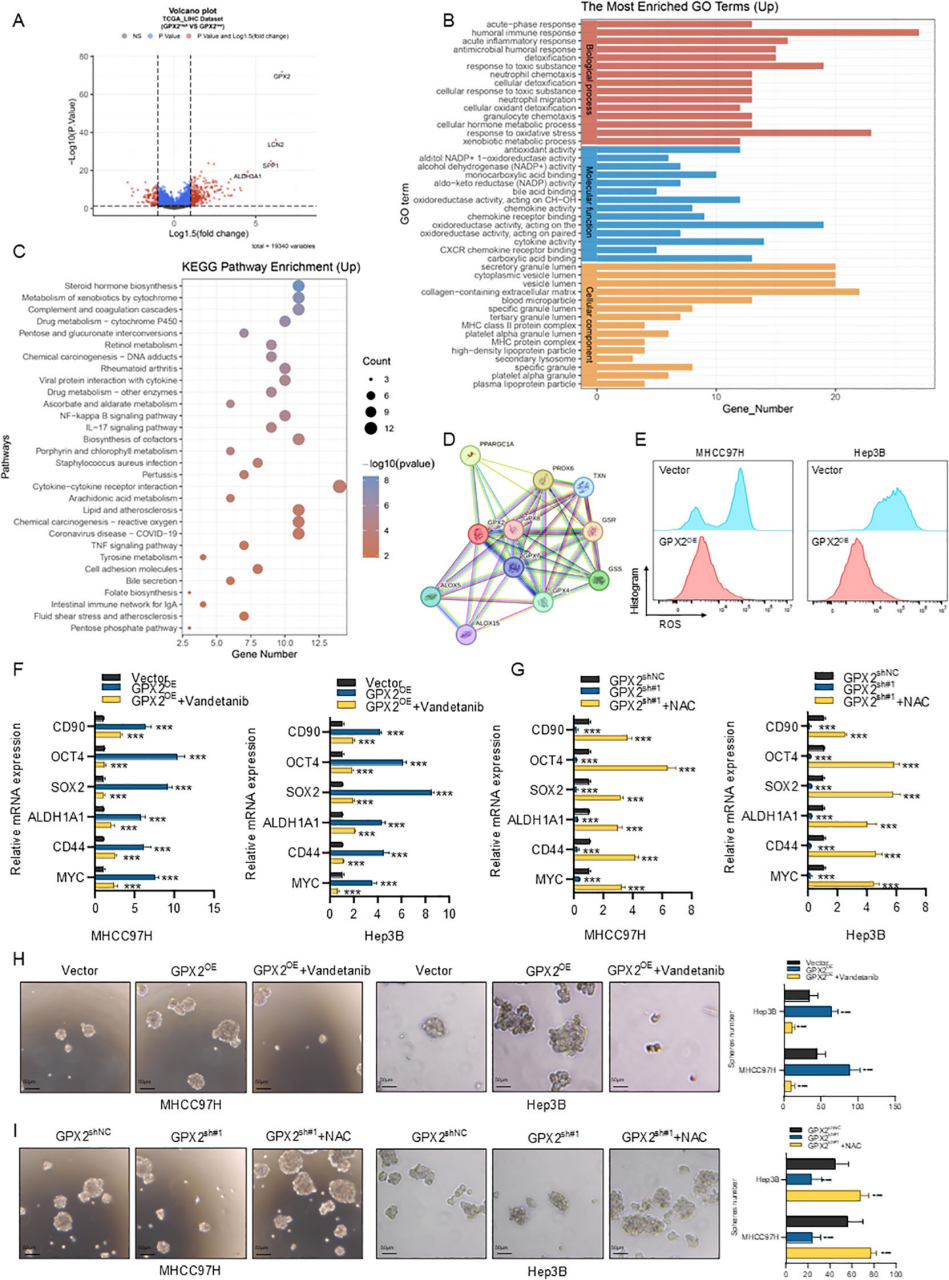
GPX2 promotes hepatocellular carcinoma stemness via the ROS–MYC signaling axis. **(A)** Volcano plot showing differentially expressed genes (DEGs) between GPX2-high and GPX2-low groups in the TCGA-LIHC dataset. **(B, C)** GO **(B)** and KEGG **(C)** enrichment analyses of upregulated DEGs in GPX2-high tumors. **(D)** Protein–protein interaction (PPI) network of GPX2 and related redox-associated proteins. **(E)** Intracellular ROS levels in MHCC97H and Hep3B cells. **(F)** Expression of stemness-associated markers in control (Vector), GPX2-overexpressing, and GPX2-overexpressing cells treated with the ROS agonist Vandetanib. **(G)** Expression of stemness-associated markers in control (shNC), GPX2-knockdown, and GPX2-knockdown cells treated with the ROS scavenger NAC. **(H)** Sphere-forming capacity of Vector, GPX2-overexpressing, and GPX2-overexpressing cells with Vandetanib treatment. **(I)** Sphere-forming capacity of shNC, GPX2-knockdown, and GPX2-knockdown cells with NAC treatment.

We next sought to elucidate the molecular mechanisms through which GPX2 regulates cancer stem cell–like properties in hepatocellular carcinoma. Patients in the TCGA-LIHC dataset were stratified into GPX2 high- and low-expression groups, and differentially expressed genes (DEGs) between the two cohorts were identified using DESeq2, with thresholds set at P < 0.01 and |log_2_FC| > 1.5. The results were visualized in a volcano plot (**Figure 5A**). GO and KEGG enrichment analyses of the DEGs revealed that high GPX2 expression was primarily associated with oxidative stress, reactive oxygen species (ROS)–related pathways, and immune-associated signaling (23) (**Figures 5B, C**). Furthermore, a protein–protein interaction (PPI) network constructed via the STRING database demonstrated significant associations between GPX2 and other redox-related proteins, including GPX8, GPX7, GPX4, PRDX6, and GSR (**Figure 5D**). Based on these findings, we hypothesize that GPX2, similar to other members of the GPX family, may regulate intracellular ROS homeostasis.

To validate this hypothesis, we assessed the intracellular reactive oxygen species (ROS) levels in two GPX2-overexpressing HCC cell lines alongside their respective controls. Flow cytometry analysis revealed that GPX2 overexpression led to a significant reduction in cellular ROS levels (**Figure 5E**). Given that prior studies have established the critical role of the equilibrium between ROS and antioxidants in regulating cancer stem cell plasticity, we further investigated whether GPX2 influences HCC stemness via ROS modulation (24). To this end, we employed Vandetanib, a ROS agonist, and N-acetylcysteine (NAC), a ROS inhibitor. qPCR analysis of cancer stem cell markers demonstrated that GPX2 overexpression markedly enhanced the stem-like properties of HCC cells. Notably, this effect was significantly reversed by the exogenous administration of Vandetanib (**Figure 5F**). Conversely, the diminished expression of stemness markers resulting from GPX2 knockdown was effectively rescued by the addition of NAC (**Figure 5G**). Consistent with these findings, results from sphere formation assays provided further support for our conclusion. The exogenous administration of Vandetanib significantly counteracted the increase in sphere formation induced by GPX2 overexpression (**Figure 5H**). Conversely, N-acetylcysteine (NAC) treatment promoted spherogenesis in GPX2-knockdown HCC cells (**Figure 5I**). Collectively, these results elucidate a potential mechanism wherein GPX2 modulates the cancer stem cell-like properties of HCC, likely through the regulation of intracellular ROS levels.

**Figure 6:**
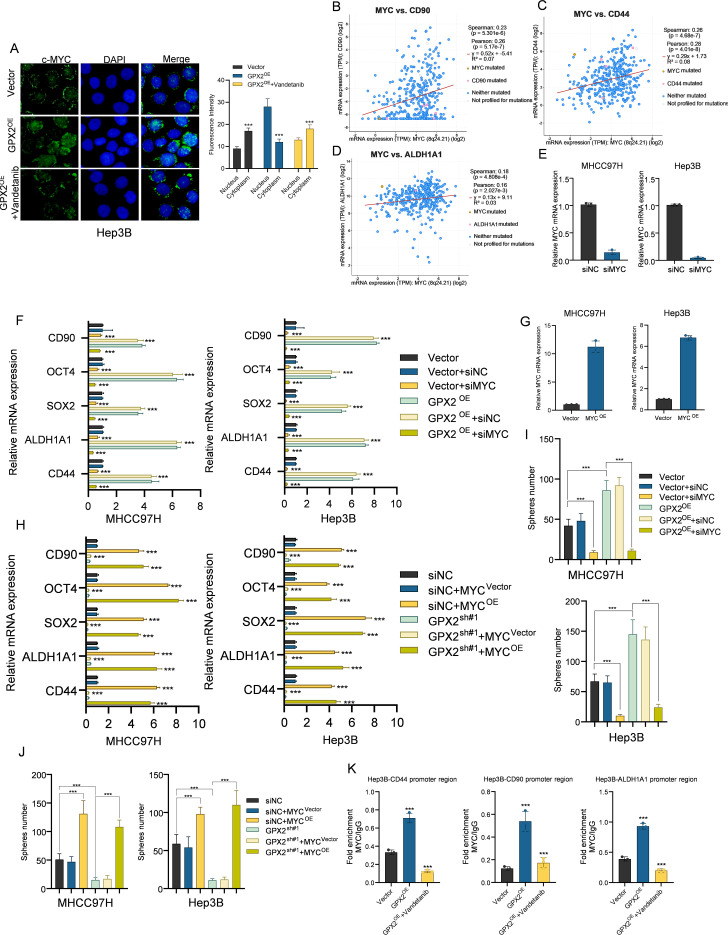
GPX2 promotes hepatocellular carcinoma stemness via the ROS–MYC signaling axis. **(A)** Immunofluorescence images showing intracellular localization of MYC in Vector, GPX2-overexpressing, and GPX2-overexpressing cells treated with the ROS agonist Vandetanib. **(B–D)** Correlation analysis between MYC and stemness-associated markers (CD90, CD44, ALDH1A1) in the TCGA-LIHC dataset. **(E)** Knockdown efficiency of MYC in MHCC97H and Hep3B cells. **(F)** Rescue experiments in Vector and GPX2-overexpressing cells transfected with siNC or siMYC, showing mRNA expression of stemness-associated markers. **(G)** Overexpression efficiency of MYC in MHCC97H and Hep3B cells. **(H)** Rescue experiments in Vector and GPX2-overexpressing cells transfected with MYC-Vector or MYC-overexpression plasmids, showing mRNA expression of stemness-associated markers. **(I)** Sphere formation assays comparing Vector, GPX2-overexpressing cells transfected with siNC or siMYC. **(J)** Sphere formation assays comparing Vector, GPX2-overexpressing cells transfected with MYC-Vector or MYC-overexpression plasmids. **(K)** ChIP-qPCR Results of MYC Binding to the Promoter Regions of CD44, CD90, and ALDH1A1 in Hep3B Cells. *** P<0.001.

We next sought to elucidate the specific mechanism underlying the GPX2-ROS axis in the regulation of HCC cancer stem cell properties. Prior studies have linked intracellular ROS levels to the activity and subcellular localization of the MYC proto-oncogene. We therefore hypothesized that in HCC cells, GPX2 might influence the nuclear-cytoplasmic distribution of c-MYC via ROS modulation. To investigate this, we performed cellular immunofluorescence staining, using an anti-c-MYC antibody to probe for intracellular c-MYC and DAPI for nuclear counterstaining. The results revealed that GPX2 overexpression led to a significant accumulation of c-MYC within the nucleus, an effect that was markedly diminished upon treatment with Vandetanib (**Figure 6A**). Given that MYC functions as a transcription factor for stemness-related genes, we further examined the correlation between the expression of MYC and established HCC stem cell markers, such as CD90, CD44, and ALDH1A1, within the TCGA-LIHC dataset (**Figures 6B–D**). This analysis revealed a significant positive correlation, implicating MYC as a potential key factor in the enhancement of stem-like properties observed in GPX2-high cells. To validate this conjecture, we designed a plasmid for MYC overexpression and a small interfering RNA (siRNA) targeting MYC (**Figures 6E, G**). The respective efficiencies of overexpression and knockdown were subsequently confirmed via qPCR.

Subsequently, we performed MYC knockdown experiments in GPX2-overexpressing HCC cells. qPCR results demonstrated that silencing MYC significantly abrogated the upregulation of stemness-related genes previously induced by GPX2. Concomitantly, we conducted rescue experiments by overexpressing MYC in GPX2-knockdown cells. These results confirmed that ectopic MYC expression could effectively rescue the diminished expression of stemness genes caused by GPX2 deficiency (**Figures 6F, H**). These molecular findings were further corroborated by sphere formation assays. The enhanced spherogenesis resulting from GPX2 overexpression was reversed by MYC knockdown, whereas the impaired sphere-forming ability in GPX2-knockdown cells was restored by MYC overexpression (**Figures 6I, J**). Based on previous findings confirming that GPX2 regulates nuclear translocation of MYC through modulation of ROS levels, this study further investigated whether altering GPX2 or ROS levels affects the binding affinity of MYC to promoters of stemness-related genes. Analysis via the JASPAR database confirmed that the promoter regions of CD44, CD90, and ALDH1A1 all contain MYC binding sites. Specific primers targeting the promoter regions of these genes were designed and synthesized. Chromatin immunoprecipitation (ChIP) assays revealed that overexpression of GPX2 significantly enhanced the binding of MYC to the promoters of CD44, CD90, and ALDH1A1, whereas treatment with a ROS inducer markedly attenuated this binding. These results indicate that the GPX2-ROS-MYC signaling axis transcriptionally regulates the expression of tumor stemness-associated genes (**Figure 6K**; **Supplementary Figure 5**).

In summary, through these pivotal rescue experiments centered on MYC, we have substantiated that GPX2 sustains the cancer stem cell-like properties of HCC through the ROS-MYC signaling axis.

### 3.6 GPX2 promotes immune modulation in hepatocellular carcinoma through MYC-dependent induction of LGALS1 in B cells

**Figure 7:**
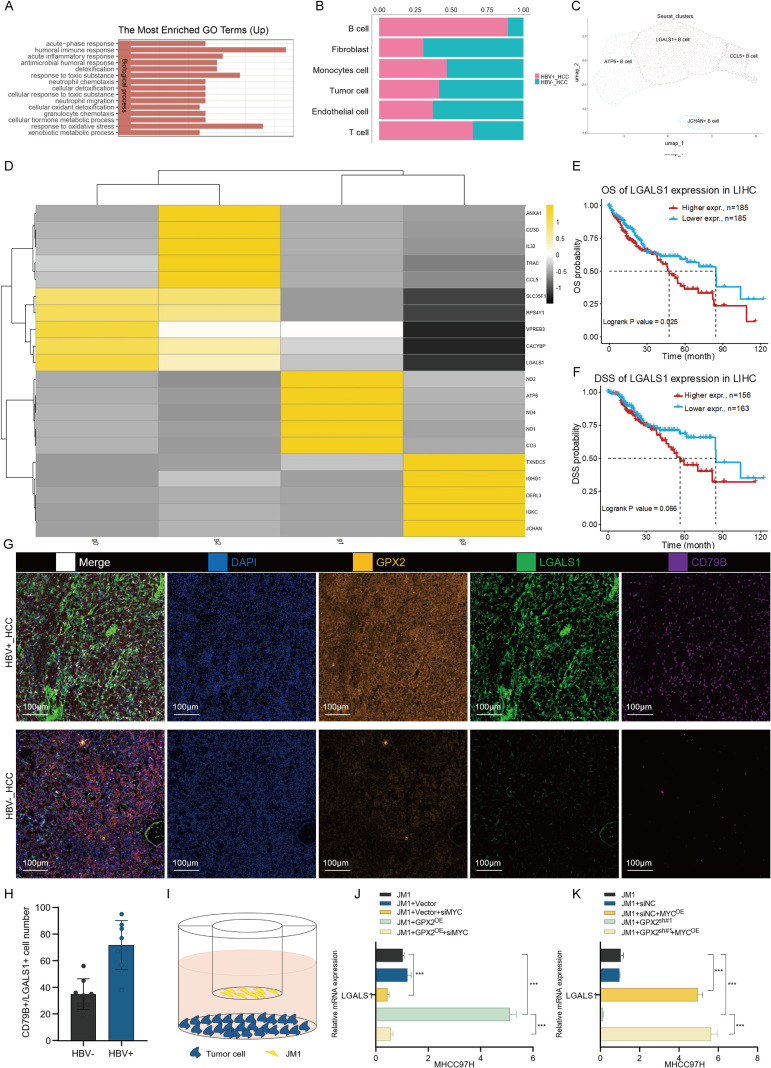
GPX2 promotes immune modulation in hepatocellular carcinoma through MYC-dependent induction of LGALS1 in B cells. **(A)** GO enrichment analysis (Biological Process) of genes upregulated in GPX2-high patients from the TCGA-LIHC cohort. **(B)** Bar plot showing proportions of different immune cell types in HBV-negative and HBV-positive HCC. **(C)** UMAP visualization of scRNA-seq data from B cells across all HCC samples, color-coded by subpopulation identity. **(D)** Heatmap of representative marker genes for major B-cell clusters. **(E, F)** Kaplan–Meier curves comparing overall survival **(E)** and disease-specific survival **(F)** between LGALS1-high and LGALS1-low patients in the TCGA-LIHC cohort. **(G)** Multiplex immunohistochemistry (mIHC) of HBV-negative and HBV-positive HCC tissues (blue, DAPI; yellow, GPX2; green, LGALS1; purple, CD79B). **(H)** Quantification of CD79B^+^LGALS1^+^ B cells per microscopic field. **(I)** Schematic illustration of the co-culture system for JM1 B cells and tumor cells. **(J)** qPCR analysis of LGALS1 expression in JM1 cells co-cultured with MHCC97H Vector, GPX2-overexpressing, or MYC-silenced tumor cells. **(K)** qPCR analysis of LGALS1 expression in JM1 cells co-cultured with MHCC97H shNC, GPX2-knockdown, or MYC-overexpressing tumor cells. *** P<0.001.

Accumulating evidence indicates that cancer stem cells (CSCs) not only play a pivotal role in tumor growth and proliferation but also facilitate immune evasion by remodeling the tumor microenvironment. For instance, research by Steven M. Pollard’s group revealed that in glioblastoma, CSCs undergo epigenetic reprogramming to promote the recruitment of myeloid cells, thereby fostering a highly infiltrated, immunosuppressive myeloid milieu that enables immune escape and tumor progression (25). GO enrichment analysis of GPX2-high tumor cells revealed that this population is primarily associated with immune regulatory signaling pathways (**Figure 7A**). Guided by these precedents, we retrospectively analyzed the cellular composition of the immune microenvironment in both HBV-negative and HBV-positive hepatocellular carcinoma (HCC). This comparative analysis led to a striking observation: the infiltration of B cells was profoundly elevated in HBV-positive HCC, reaching a level nearly fivefold higher than that in their HBV-negative counterparts (**Figure 7B**). This unique phenomenon captured our attention and warranted further investigation.

We next performed reclustering and annotation of the B cell compartment. As shown in the results, B cells segregated into four major subclusters, which were subsequently annotated as ATP6^+^ B cells, LGALS1^+^ B cells, CCL5^+^ B cells, and JCHAIN^+^ B cells (**Figures 7C, D**). Notably, LGALS1 has been reported as a marker of poor prognosis in hepatocellular carcinoma (**Figures 7E, F**). To investigate its clinical significance, patients from the TCGA-LIHC cohort were stratified into high- and low-expression groups based on LGALS1 expression, and overall survival (OS) and disease-specific survival (DSS) were analyzed (**Figures 7E, F**). The survival curves indicated that patients with higher LGALS1 expression exhibited significantly worse outcomes. To validate these findings in clinical samples, we collected HBV-negative and HBV-positive HCC tissues and performed multiplex immunohistochemistry (mIHC). Tumor cells and B cells were labeled with antibodies against GPX2, LGALS1, and CD79B, followed by quantitative analysis (**Figure 7G**). The results demonstrated that CD79B^+^/LGALS1^+^ B cells were markedly more abundant in HBV-positive HCC tissues compared with HBV-negative counterparts (**Figure 7H**).

To investigate the regulatory effects of GPX2^+^ tumor cells on B cells *in vitro*, we established a tumor cell–B cell co-culture system (**Figure 6I**). In this model, JM1 cells, an immortalized B-cell lymphoma line, were used as the primary surrogate for B cells. As illustrated in **Figure 7I**, tumor cells were seeded in the lower compartment of the co-culture system, while JM1 cells were maintained in suspension in the upper chamber. After 72 hours of co-culture, JM1 cells were collected, and RNA was extracted for qPCR analysis of LGALS1 expression. The results showed that JM1 cells co-cultured with control MHCC97H cells exhibited only a modest increase in LGALS1 levels (**Figure 7J**). In contrast, co-culture with GPX2-overexpressing MHCC97H cells significantly upregulated LGALS1 mRNA in JM1 cells, an effect that was abrogated by MYC knockdown (**Figure 7J**). Conversely, JM1 cells co-cultured with GPX2-silenced MHCC97H cells displayed markedly reduced LGALS1 expression, whereas enforced MYC overexpression rescued this inhibitory effect (**Figure 7K**).

In summary, our experimental findings provide preliminary evidence that GPX2 not only enhances stemness properties within hepatocellular carcinoma cells but also modulates the immune microenvironment by activating LGALS1 expression in B cells.

### 3.7 GPX2–CCL26 axis promotes LGALS1^+^ B-cell accumulation and immune evasion in HBV-positive HCC

**Figure 8:**
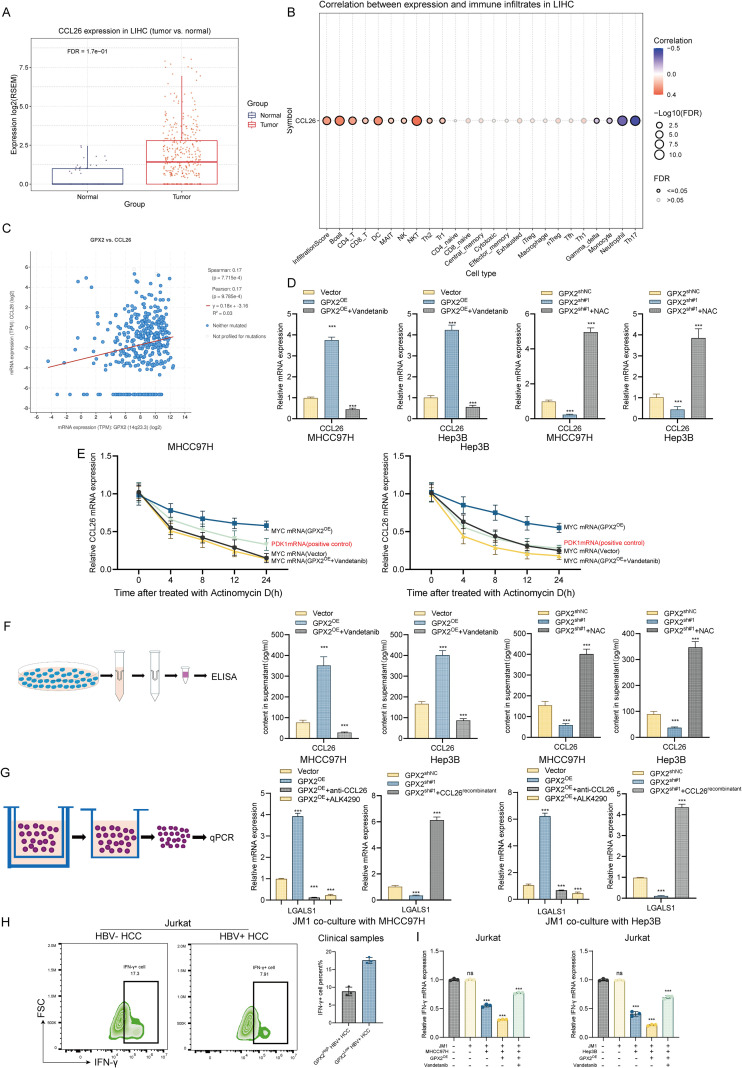
GPX2–CCL26 axis promotes LGALS1^+^ B-cell accumulation and immune evasion in HBV-positive HCC. **(A)** CCL26 expression in tumor versus normal liver tissues from the TCGA-LIHC dataset. **(B)** Correlation between CCL26 expression and immune cell infiltration estimated by ImmuCellAI, showing positive associations with B cells, CD4^+^ T cells, CD8^+^ T cells, dendritic cells (DCs), and natural killer (NK) cells, and negative correlations with monocytes, neutrophils, and Th17 cells. **(C)** Correlation analysis between GPX2 and CCL26 expression in the TCGA-LIHC dataset. **(D)** qPCR analysis of CCL26 mRNA expression in MHCC97H and Hep3B cells with GPX2 overexpression or knockdown, with or without Vandetanib (ROS agonist) or NAC (ROS scavenger). **(E)** CCL26 mRNA Stability Analysis in MHCC97H and Hep3B Cell Lines via Half-life Measurement Assay. **(F)** LISA quantification of secreted CCL26 levels in culture supernatants. **(G)** Co-culture assays of JM1 B cells with MHCC97H cells: LGALS1 expression changes upon GPX2 modulation with or without recombinant CCL26, CCL26 neutralization, or CCR3 inhibition. **(H)** Flow Cytometric Quantification and Statistical Representation of IFN-γ Expression in Hepatoma Cells Following Co-culture with Jurkat Cells in Clinical Specimens. **(I)** qPCR of IFN-γ Expression in MHCC97H and Hep3B Cells Following Co-culture with Jurkat Cells. *** P<0.001.

To further investigate the mechanism by which GPX2-high hepatocellular carcinoma cells induce LGALS1 upregulation in B cells, we focused on CCL26, a known B-cell–stimulating factor. Previous studies have shown that CCL26 promotes B-cell differentiation, and that tumor-associated fibroblast–derived CCL26 can engage CCR3 on the B-cell surface to drive their differentiation into tumor-associated B cells, thereby facilitating immune evasion (26, 27). Analysis of the TCGA-LIHC dataset revealed that CCL26 expression was markedly elevated in tumor tissues compared with normal liver tissues (**Figure 8A**). To explore the potential immunological implications, we applied ImmuCellAI to estimate immune cell infiltration across patients and correlated infiltration scores with CCL26 expression. The results showed that CCL26 levels were significantly associated with immune infiltration in HCC. Specifically, CCL26 expression correlated positively with B cells, CD4^+^ T cells, CD8^+^ T cells, dendritic cells (DCs), and natural killer (NK) cells, while exhibiting significant negative correlations with monocytes, neutrophils, and Th17 cells (**Figure 8B**).

Correlation analysis of the TCGA-LIHC dataset revealed a positive association between GPX2 and CCL26 expression, suggesting that GPX2 may regulate CCL26 through modulation of intracellular ROS levels (**Figure 8C**). To test this hypothesis, we examined CCL26 mRNA levels in MHCC97H and Hep3B cells with GPX2 overexpression or knockdown, with or without treatment by the ROS agonist Vandetanib or the ROS scavenger N-acetylcysteine (NAC). The results showed that GPX2 overexpression markedly increased CCL26 mRNA expression, an effect that was reversed by Vandetanib (**Figure 8D**). Conversely, GPX2 knockdown reduced CCL26 mRNA levels, which could be rescued by NAC treatment (**Figure 8D**). Subsequently, we measured the mRNA half-life of CCL26 in MHCC97H and Hep3B cells. The results demonstrated that overexpression of GPX2 prolonged the mRNA half-life of CCL26, whereas knockdown of GPX2 shortened it (**Figure 8E**). These findings indicate that GPX2 protects CCL26 transcripts from ROS-mediated suppression. ELISA analysis of secreted CCL26 levels yielded results consistent with qPCR, further supporting the notion that GPX2 enhances CCL26 secretion through stabilization of its mRNA (**Figure 8F**).

To determine whether CCL26 mediates the effects of GPX2-high tumor cells on B-cell function, we performed co-culture assays supplemented with recombinant CCL26, neutralizing antibodies against CCL26, or the CCR3 inhibitor. qPCR analysis revealed that the GPX2-induced upregulation of LGALS1 in JM1 B cells was abolished by either CCL26 neutralization or CCR3 inhibition (**Figure 8G**). In contrast, exogenous CCL26 supplementation reversed the reduction in LGALS1 expression observed upon GPX2 knockdown (**Figure 8G**). Subsequently, we collected tumor tissues from clinical surgical samples, performed digestion and filtration to isolate tumor cells, and co-cultured them with JM1 cells. The treated JM1 cells were then co-cultured with Jurkat cells, and IFN-γ expression in Jurkat cells was assessed (**Figure 8H**). The results revealed that JM1 cells induced by HBV^+^ HCC cells significantly reduced IFN-γ expression levels in Jurkat cells upon co-culture. These findings were further validated in both MHCC97H and Hep3B cell lines (**Figure 8I**).

Collectively, these results demonstrate that GPX2^+^ tumor cells stabilize CCL26 mRNA to enhance CCL26 secretion, thereby driving B-cell differentiation and promoting LGALS1 expression, ultimately contributing to immune evasion in HBV-related HCC.

### 3.8 GPX2 drives tumor progression *in vivo* and B-Cell targeting potentiates anti-PD1-mediated elimination of GPX2-High expressing cells

**Figure 9:**
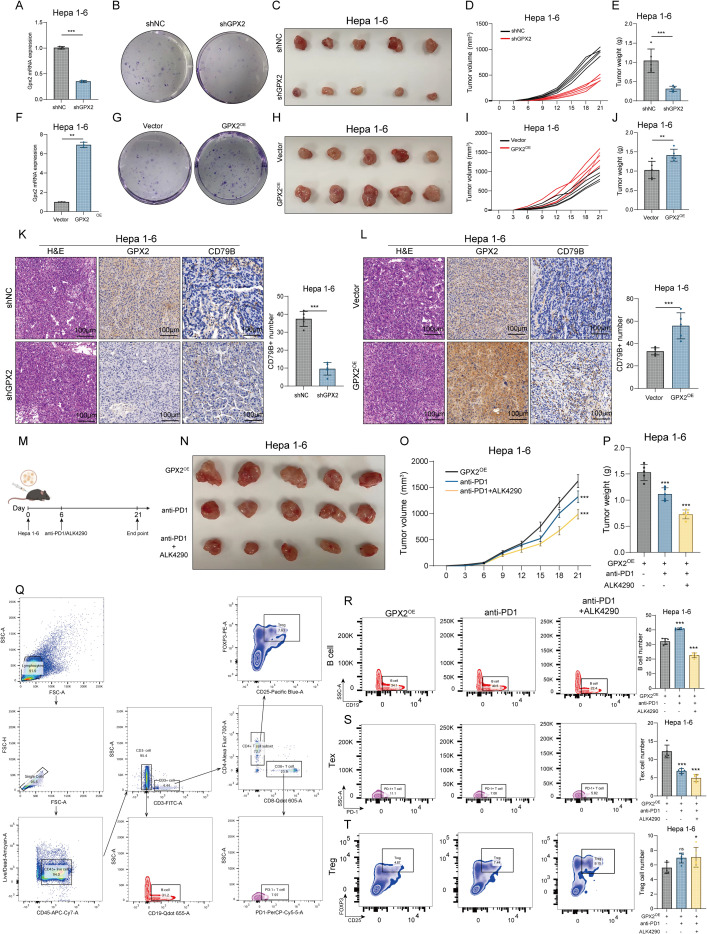
GPX2 drives tumor progression *in vivo* and B-Cell targeting potentiates anti-PD1-mediated elimination of GPX2-High expressing cells. **(A)** qPCR Detection of GPX2 Knockdown Efficiency in Hepa1–6 Cells. **(B)** Colony Formation Assay (96h) of Hepa1–6 Cells with GPX2 Knockdown and Control Groups. **(C)** Subcutaneous Tumor Xenograft Experiment Results of Hepa1–6 Cells with GPX2 Knockdown and Control Groups. **(D)** Tumor Growth Curves from the Subcutaneous Xenograft Assay of Hepa1–6 Cells with GPX2 Knockdown versus Control Groups. **(E)** Tumor Weight in Subcutaneous Xenograft Assay of Hepa1–6 Cells with GPX2 Knockdown versus Control Groups. **(F)** qPCR Detection of GPX2 Overexpression Efficiency in Hepa1–6 Cells. **(G)** Colony Formation Assay (96h) of Hepa1–6 Cells with GPX2 Overexpression and Control Groups. **(H)** Subcutaneous Tumor Xenograft Experiment Results of Hepa1–6 Cells with GPX2 Overexpression and Control Groups. **(I)** Tumor Growth Curves from the Subcutaneous Xenograft Assay of Hepa1–6 Cells with GPX2 Overexpression versus Control Groups. **(J)** Tumor Weight in Subcutaneous Xenograft Assay of Hepa1–6 Cells with GPX2 Overexpression versus Control Groups. **(K)** H&E and IHC (GPX2 and CD79B) Staining Results with Statistical Analysis of Subcutaneous Tumors from Hepa1–6 Cells with GPX2 Knockdown versus Control Groups. **(L)** H&E and IHC (GPX2 and CD79B) Staining Results with Statistical Analysis of Subcutaneous Tumors from Hepa1–6 Cells with GPX2 Overexpression versus Control Groups. **(M)** Schematic Diagram of the Animal Experiment Strategy. **(N)** Experimental Results of Subcutaneous Tumor Xenograft Assay with GPX2 Overexpression, Anti-PD1 Therapy, and Combination Treatment. **(O)** Tumor Growth Curves from Subcutaneous Xenograft Assays of GPX2 Overexpression, Anti-PD1 Therapy, and Combination Treatment. **(P)** Tumor Weight in Subcutaneous Xenograft Assay of GPX2 Overexpression, Anti-PD1 Therapy, and Combination Treatment. **(Q)** Flow Cytometry Analysis of Different Cell Sorting Strategies. **(R)** Analysis and Statistics of Infiltrating B Cells in GPX2 Overexpression, Anti-PD1 Therapy, and Combination Treatment. **(S)** Analysis and Statistics of Infiltrating Tex Cells in GPX2 Overexpression, Anti-PD1 Therapy, and Combination Treatment. **(T)** Analysis and Statistics of Infiltrating Treg Cells in GPX2 Overexpression, Anti-PD1 Therapy, and Combination Treatment. * represents P<0.05, ** represents P<0.01, *** represents P<0.001.

Next, we validated the above findings through *in vivo* animal experiments. First, we constructed GPX2-knockdown and control (shNC) cells as well as GPX2-overexpressing and control (Vector) cells using the C57-derived Hepa1–6 cell line, and confirmed the knockdown and overexpression efficiency by qPCR (**Figures 9A, F**). Subsequently, Hepa1–6 cells (1 × 10^6^ cells per mouse) were subcutaneously injected into C57 mice. Tumor growth was monitored every three days, and the mice were euthanized at day 21 to harvest tumors. The results showed that GPX2 knockdown significantly inhibited the growth of Hepa1–6 cells *in vivo*, whereas GPX2 overexpression markedly promoted tumor growth (**Figures 9B–E, G–J**). Collected tumor tissues were subjected to H&E and immunohistochemical (IHC) staining. The results indicated that GPX2 significantly promoted the infiltration of B cells (CD79B-positive cells) within the tumors (**Figures 9K, L**).

Based on the observation that ALK4290 effectively inhibited GPX2-induced B cell activation in *in vitro* models, we designed anti-PD1 monotherapy and combination therapy (anti-PD1 + ALK4290) experiments in GPX2-overexpressing tumors(**Figure 9M**). The results demonstrated that ALK4290, a B cell-targeting agent, synergistically enhanced the efficacy of anti-PD1 treatment (**Figures 9N–P**). Flow cytometry analysis further validated these findings: the GPX2-overexpression group exhibited increased B cell infiltration, and ALK4290 effectively targeted B cells. In the combination therapy group, a reduction in infiltrated exhausted T cells was observed, confirming the synergistic anti-tumor effect of the combined treatment (**Figures 9Q–T**).

**Figure 10:**
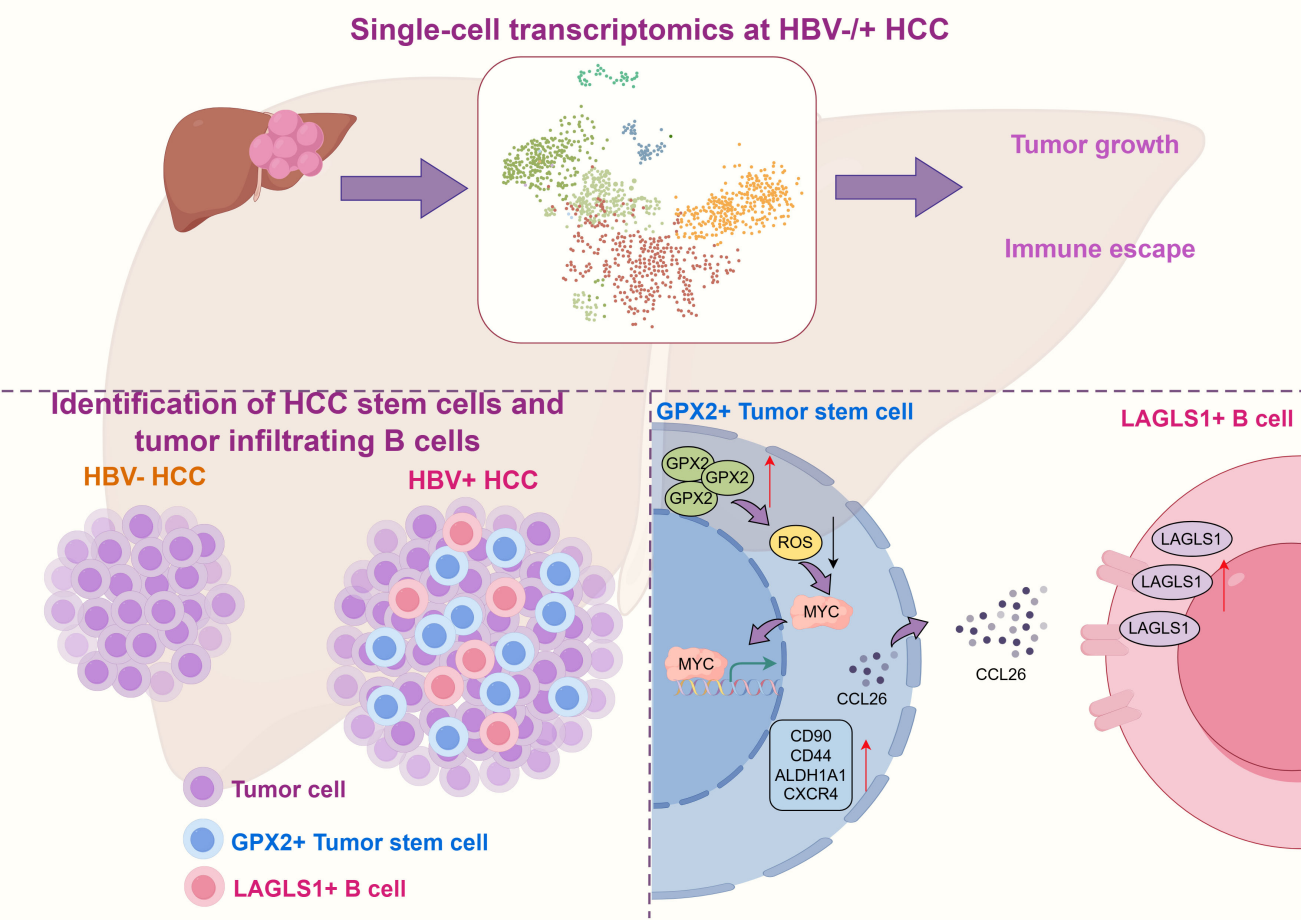
Mechanistic model of GPX2–ROS–MYC axis in HBV^+^ HCC. GPX2 reduces ROS to sustain MYC activity and CCL26 secretion, which induces immunosuppressive LGALS1^+^ B-cell differentiation.

The *in vivo* experiments conclusively demonstrated the critical role of GPX2 in promoting hepatocellular carcinoma progression. GPX2 overexpression significantly enhanced tumor growth and B-cell infiltration, while its knockdown exerted the opposite effect. Building on the *in vitro* finding that ALK4290 inhibits GPX2-mediated B-cell activation, combination therapy with anti-PD1 and ALK4290 in GPX2-overexpressing tumors revealed a potent synergistic effect. This regimen not only enhanced anti-tumor efficacy but also reshaped the tumor immune microenvironment by reducing exhausted T-cell infiltration. Together, these results establish GPX2 as a key regulator of tumor growth and immunosuppression, while validating combined B-cell targeting and PD-1 blockade as a promising therapeutic strategy for GPX2-high tumors (**Figure 10**).

## 4 Discussion

Hepatitis B virus (HBV) infection is widely recognized as a major etiological driver of hepatocellular carcinoma (HCC), conferring a 10- to 25-fold higher risk of disease compared with uninfected individuals (28). The natural course from HBV infection to HCC typically progresses through chronic hepatitis and cirrhosis before malignant transformation (29). Despite this well-established epidemiological link, the biological underpinnings of HBV-associated HCC, particularly the mechanisms governing tumor initiation and immune evasion, remain incompletely understood. This knowledge gap highlights the necessity of distinguishing HBV-related HCC from its HBV-negative counterpart in mechanistic and translational studies. Among the most intensively investigated aspects of HBV-driven hepatocarcinogenesis is the emergence and maintenance of cancer stem-like cells (CSCs) (30). Previous studies have implicated HBV in modulating stemness programs through signaling pathways such as Wnt, MAPK, Notch, and p53. Leveraging single-cell transcriptomic datasets from human HCC, our study extends this paradigm by demonstrating a significant enrichment of GPX2^+^ tumor cell populations in HBV-positive tumors, underscoring their potential role in HBV-driven oncogenesis.

GPX2, a member of the glutathione peroxidase family, has been canonically recognized for its role in regulating tumor progression by modulating intracellular levels of endogenous reactive oxygen species (ROS) (31). Historically, GPX2 has been predominantly characterized as an oncogene. For instance, in colorectal cancer (CRC), GPX2 sustains cellular proliferation and metastatic capacity by downregulating intracellular ROS (32). Furthermore, its upregulation has been shown to confer chemoresistance in lung adenocarcinoma (33). However, its specific role in the context of hepatocellular carcinoma (HCC) stem cells has remained largely unexplored. The primary substrate of GPX2 is ROS, a byproduct of cellular respiration that exerts a dichotomous influence on tumorigenesis. On one hand, ROS can promote the oxidation of various proteins, leading to functional dysregulation that fuels tumor progression (34). On the other hand, excessive ROS can induce high levels of cellular stress, thereby promoting senescence and apoptosis (34). Notably, within the cancer stem cell (CSC) paradigm, elevated ROS levels are generally considered detrimental to the maintenance of stemness.

In this study, our single-cell transcriptomic analysis first identified a significant expansion of a GPX2-high cell population specifically within HBV-positive HCC. Subsequent computational analyses, including stemness signature scoring and interrogation of the TCGA-LIHC cohort, revealed that GPX2+ tumor cells exhibit robust CSC-like characteristics. This was evidenced by the elevated expression of established HCC stem cell markers such as MYC, CD44, CXCR4, and ALDH1A1. To corroborate these findings experimentally, we demonstrated that forced overexpression of GPX2 in HCC cell lines led to a marked upregulation of these stemness markers and a concomitant enhancement of their sphere-formation capacity. To elucidate the underlying mechanism, we investigated the impact of GPX2 on intracellular ROS homeostasis. As anticipated, GPX2 overexpression resulted in a substantial decrease in cellular ROS levels, which correlated with the observed enhancement of stem-like properties. Crucially, the pro-stemness effects of GPX2 overexpression were abrogated by the exogenous administration of a ROS agonist. Conversely, the suppression of stemness induced by GPX2 knockdown was effectively rescued by the antioxidant N-acetylcysteine (NAC), firmly establishing an intricate link between GPX2, ROS modulation, and cancer stemness. In summary, our study demonstrates that the GPX2-mediated enhancement of CSC properties in HCC is mechanistically dependent on the downregulation of intracellular ROS.

To further dissect the mechanism by which GPX2 governs cancer stemness via ROS, we focused on the proto-oncogene MYC. It is well-established that the dynamic equilibrium between ROS and antioxidants is a critical determinant of cancer stem cell (CSC) properties. Furthermore, the expression and activity of MYC, a cardinal transcription factor for stemness, are known to be modulated by the cellular ROS level (35). Indeed, an antagonistic relationship between ROS levels and MYC activity has been documented. For instance, in acute myeloid leukemia (AML), ferroptosis-induced ROS upregulation was shown to suppress the BRD4/c-MYC/NRF2 axis—a critical survival and antioxidant pathway in AML (36). Another study in esophageal cancer reported that ZCCHC4 knockdown inhibits c-MYC via elevated ROS levels, a mechanism linked to tumor progression (37). Building on these precedents, we conducted a series of rescue experiments involving the modulation of c-MYC in MHCC97H and Hep3B cells. As anticipated, ectopic expression of c-MYC significantly rescued the attenuation of stemness properties caused by GPX2 knockdown. Conversely, siRNA-mediated knockdown of c-MYC abrogated the enhancement in stemness induced by GPX2 overexpression. Collectively, these findings demonstrate that GPX2 promotes an increase in the cancer stem cell-like properties of HCC by activating the ROS/c-MYC signaling axis.

Cancer stem cells (CSCs) not only intrinsically drive tumorigenesis and progression but also actively shape their immune microenvironment to facilitate immune evasion. Beyond their intrinsic signaling pathways, the extrinsic interactions between CSCs and the surrounding tumor microenvironment (TME)—mediated by direct cell-cell contact or ligand-receptor signaling with resident and infiltrating non-malignant cells—are critical drivers of tumor growth, invasiveness, and therapeutic resistance (38). For example, supernatants from cholangiocarcinoma, HCC, and glioblastoma cells cultured under CSC-enriching conditions (spheroids) exhibit elevated levels of pro-tumorigenic and macrophage-polarizing factors, including CCL2, CCL5, CSF1, GDF15, IL-13, and TGF-β (39–41). Reciprocally, tumor-associated macrophages (TAMs) can induce a CSC phenotype through soluble mediators (42). Furthermore, CSCs can subvert the anti-tumor properties of dendritic cells (DCs) by impeding their trafficking, preventing their maturation, and promoting the differentiation of tolerogenic subtypes.

Despite this mounting evidence of CSC-immune interplay across various malignancies, the specific mechanisms by which CSCs shape the immune landscape in HBV-positive HCC remain poorly characterized. We therefore conducted a differential analysis of immune cell infiltration between HBV-negative and HBV-positive HCC, which drew our attention to a pronounced disparity in the B cell population. Sub-clustering and detailed annotation of this compartment revealed a predominant population of LGALS1-expressing B cells (LGALS1+ B cells), an observation of clinical significance as high LGALS1 expression in HCC is correlated with poor prognosis. To investigate the upstream regulation of this B cell phenotype, we established a co-culture system. This experiment confirmed that GPX2-overexpressing tumor cells can potently induce the expression of LGALS1 in B cells, thereby fostering an immunosuppressive phenotype conducive to immune escape. To identify the soluble factor mediating this interaction, we analyzed GPX2-overexpressing cells and found, through ELISA and qPCR, a significant increase in both the expression and secretion of CCL26, a key cytokine involved in B cell differentiation. Crucially, targeted modulation of CCL26 in our co-culture system significantly attenuated the GPX2-driven upregulation of LGALS1 in B cells.

In summary, our study delineates a previously unrecognized role of GPX2 in HBV-associated hepatocarcinogenesis, whereby it reinforces cancer stem cell–like properties through the ROS/c-MYC axis and concurrently remodels the immune microenvironment by promoting CCL26-mediated induction of immunosuppressive LGALS1^+^ B cells. These dual functions position GPX2 as a central node linking intrinsic tumor plasticity with extrinsic immune evasion, thereby advancing our mechanistic understanding of HBV-positive HCC. Beyond its biological implications, these findings highlight GPX2 as a potential therapeutic target, not only for eradicating CSCs but also for disrupting tumor–immune crosstalk. Future investigations are warranted to explore pharmacologic strategies aimed at modulating GPX2 activity or its downstream effectors, which may offer novel avenues for precision therapy in HBV-related liver cancer.

## Data Availability

The original contributions presented in the study are included in the article/**Supplementary Material**. Further inquiries can be directed to the corresponding author.
