# Supplementary figures and images for: GPX2+ tumor cells recruit LGALS1+ B cells via CCL26-CCR3 axis to promote immunosuppression and tumor progression in hepatocellular carcinoma
Source: Front Immunol. 2026 Mar 20;17:1709855. doi: 10.3389/fimmu.2026.1709855 (PMC13047122; doi:10.3389/fimmu.2026.1709855)

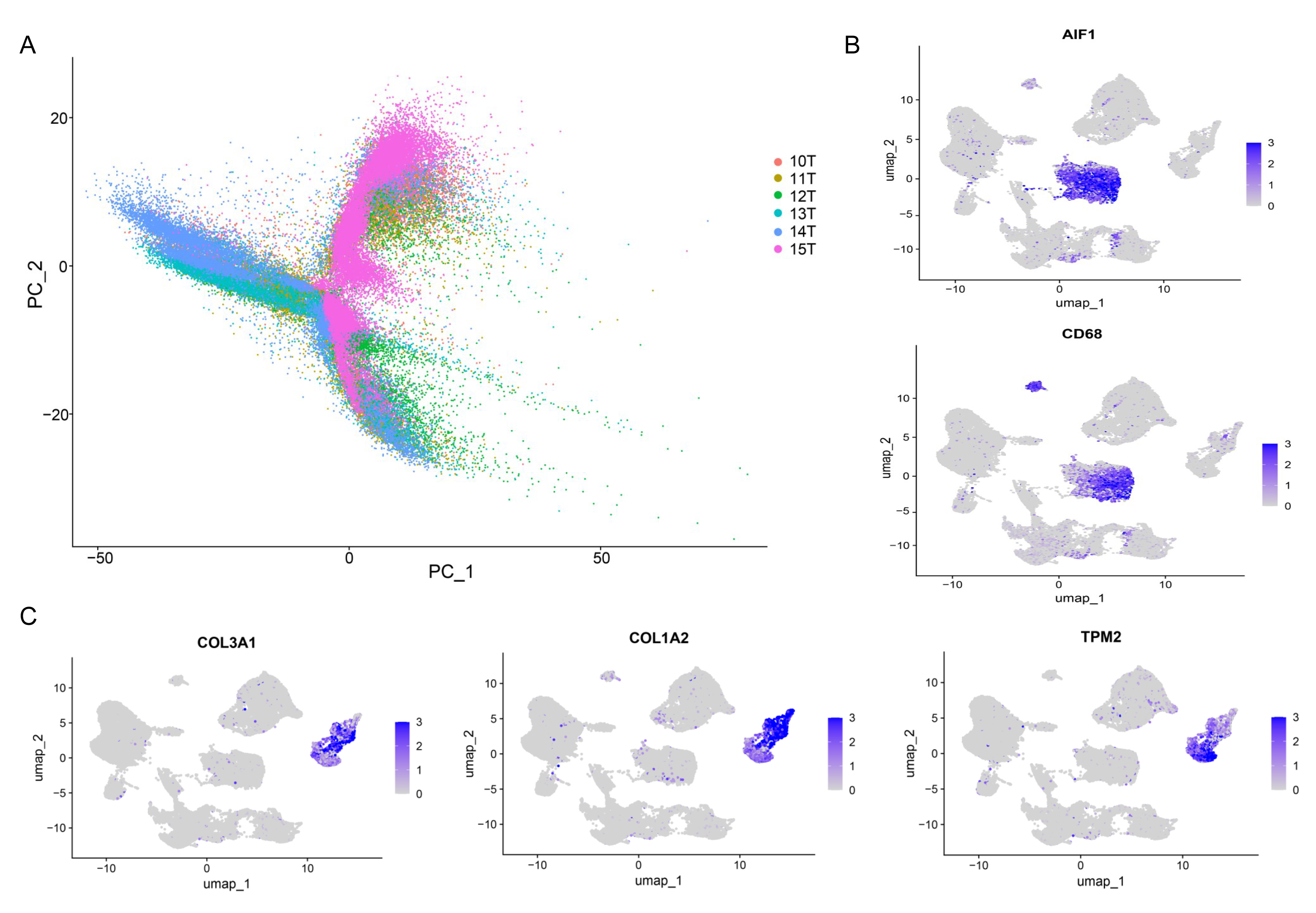

Supplement: Supplementary file 1 [file Image1.tif]

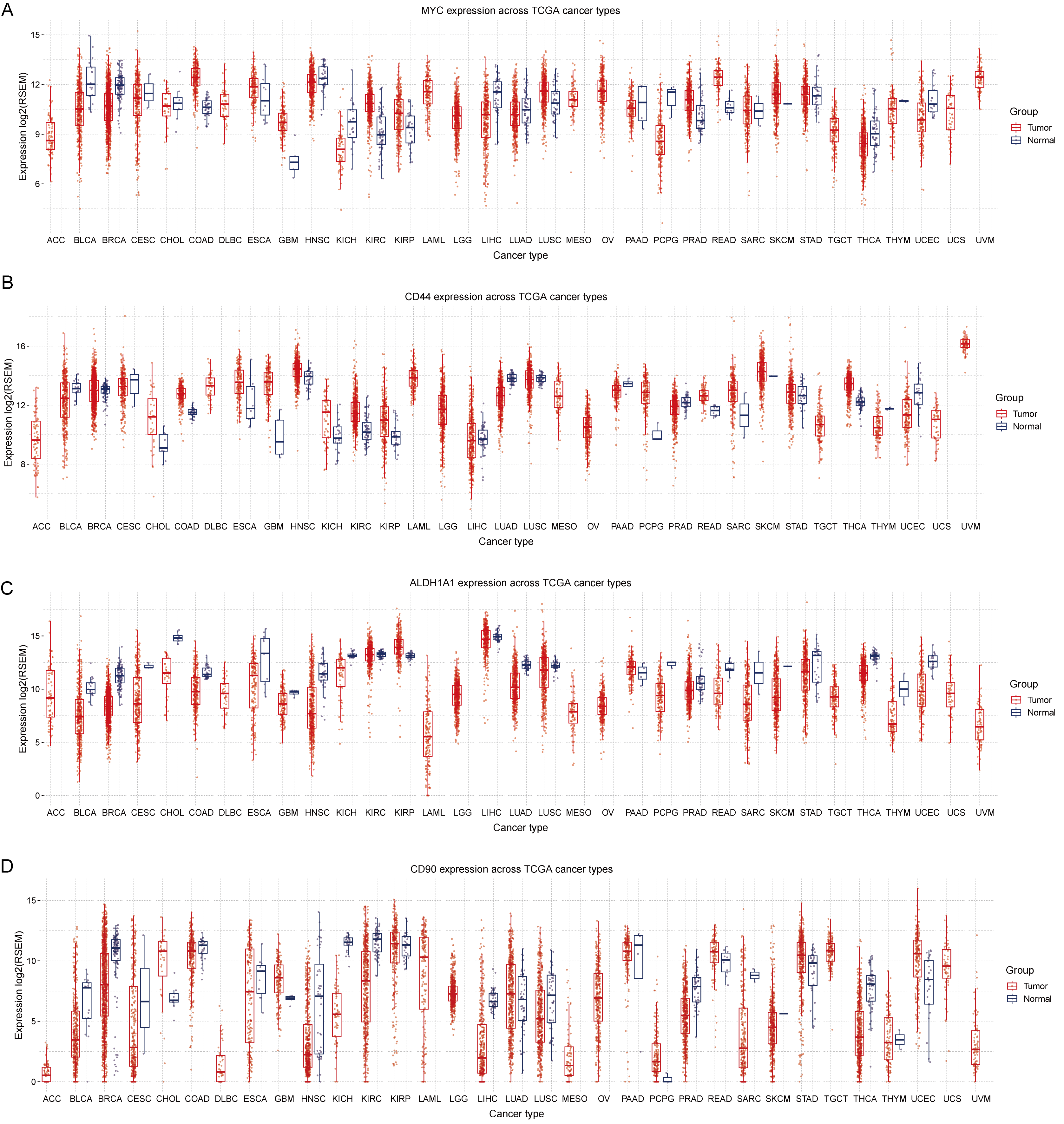

Supplement: Supplementary file 2 [file Image2.tif]

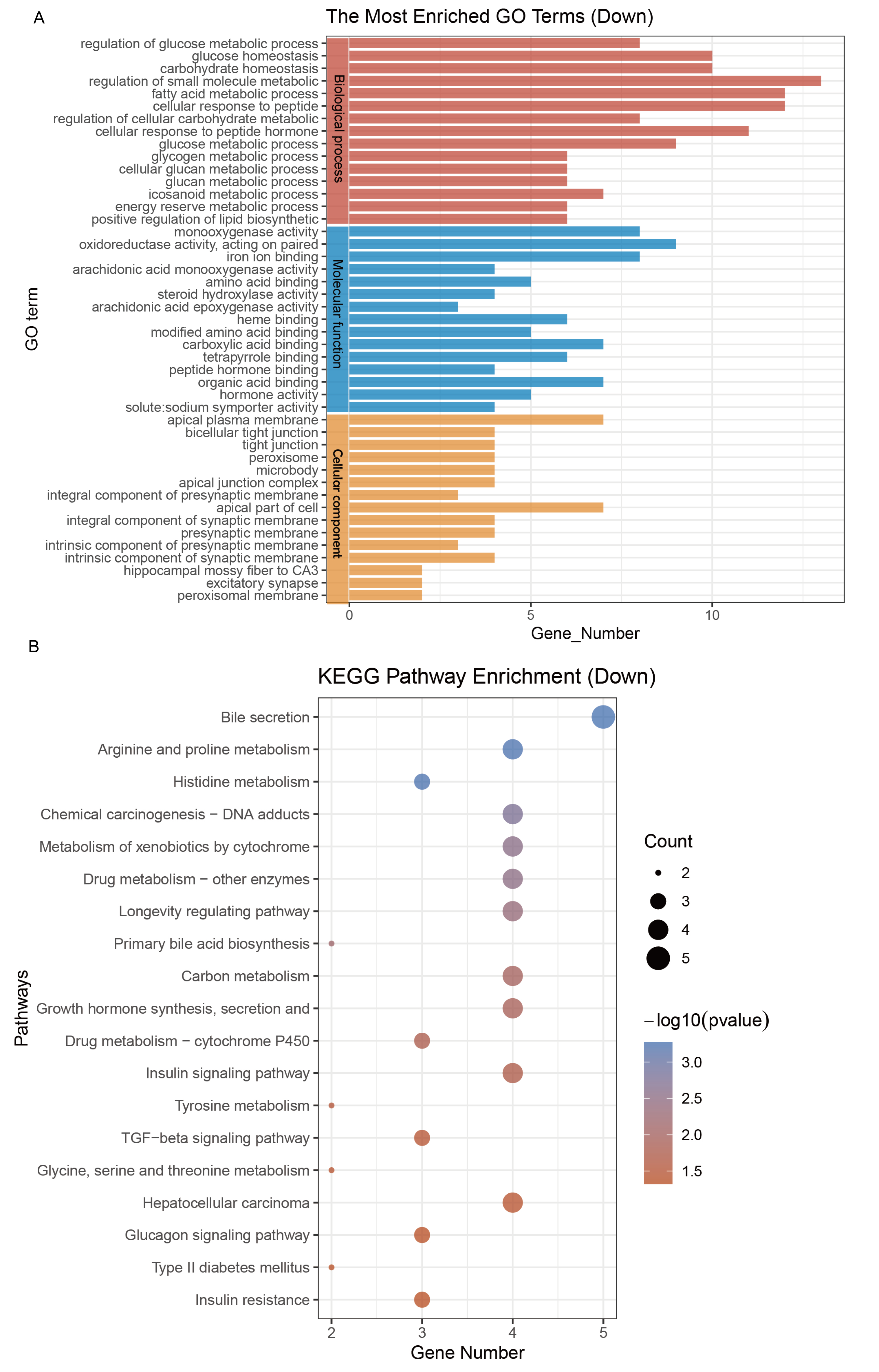

Supplement: Supplementary file 3 [file Image3.tif]

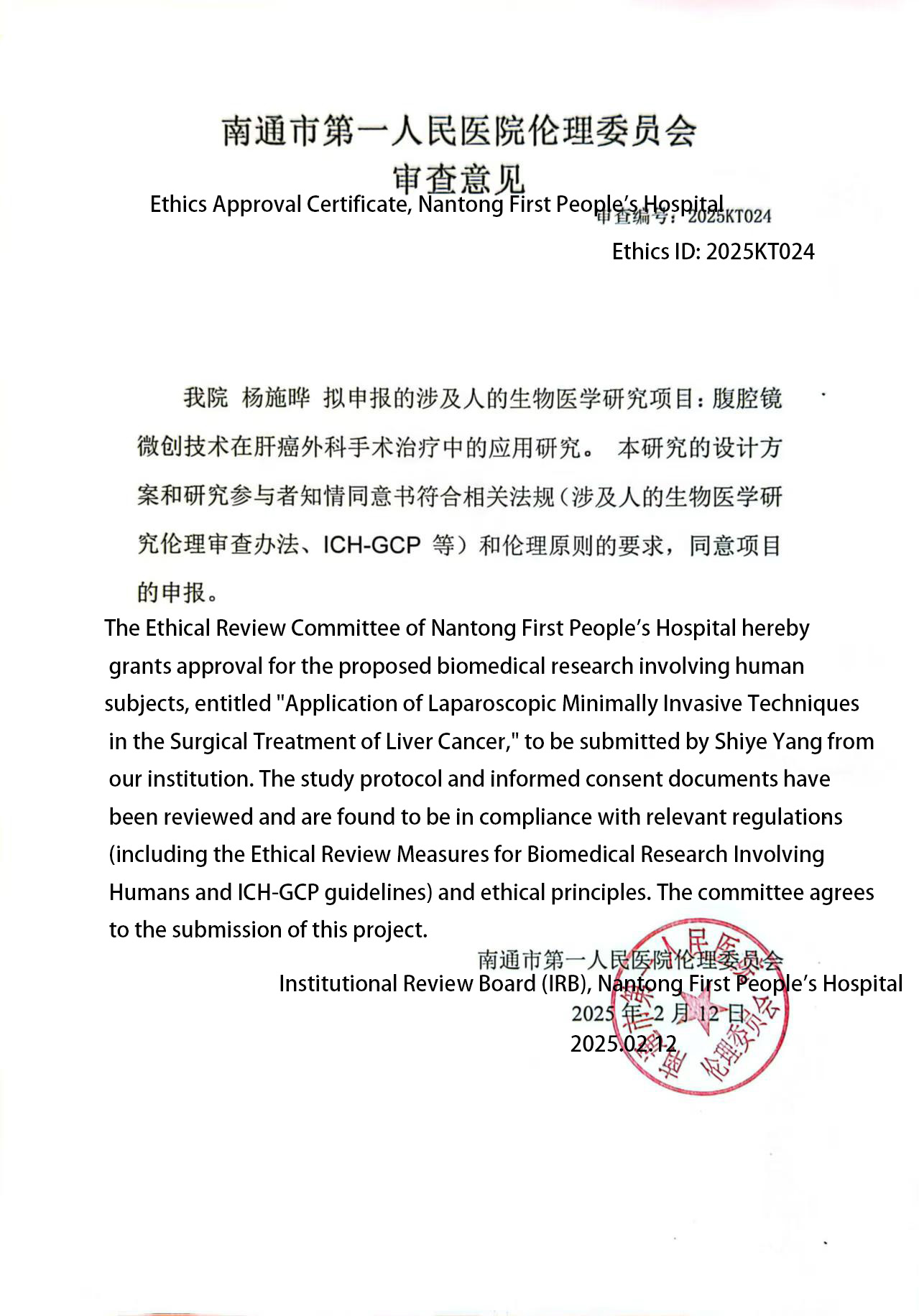

Supplement: Supplementary file 4 [file Image4.jpeg]

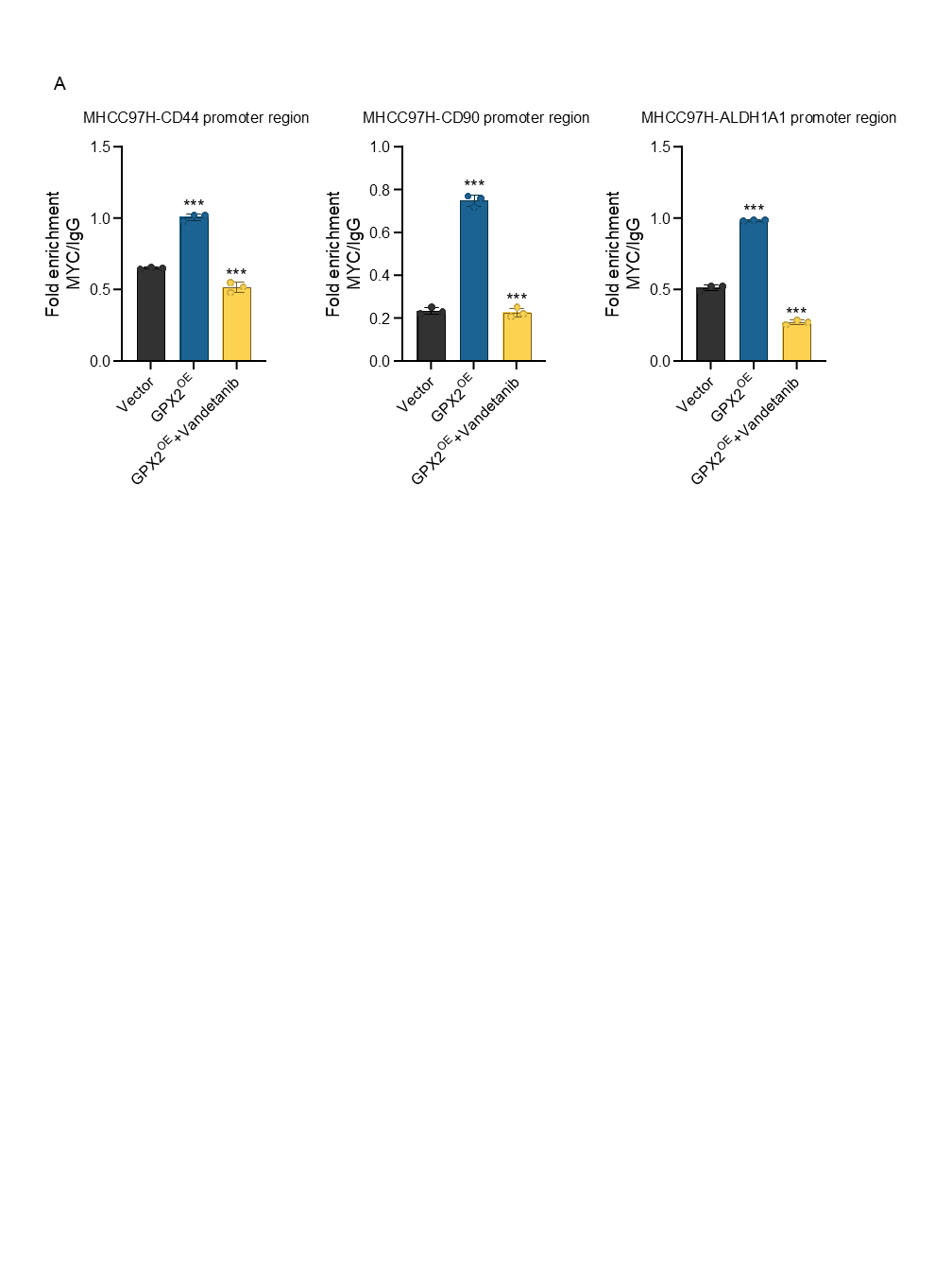

Supplement: Supplementary file 5 [file Image5.tif]

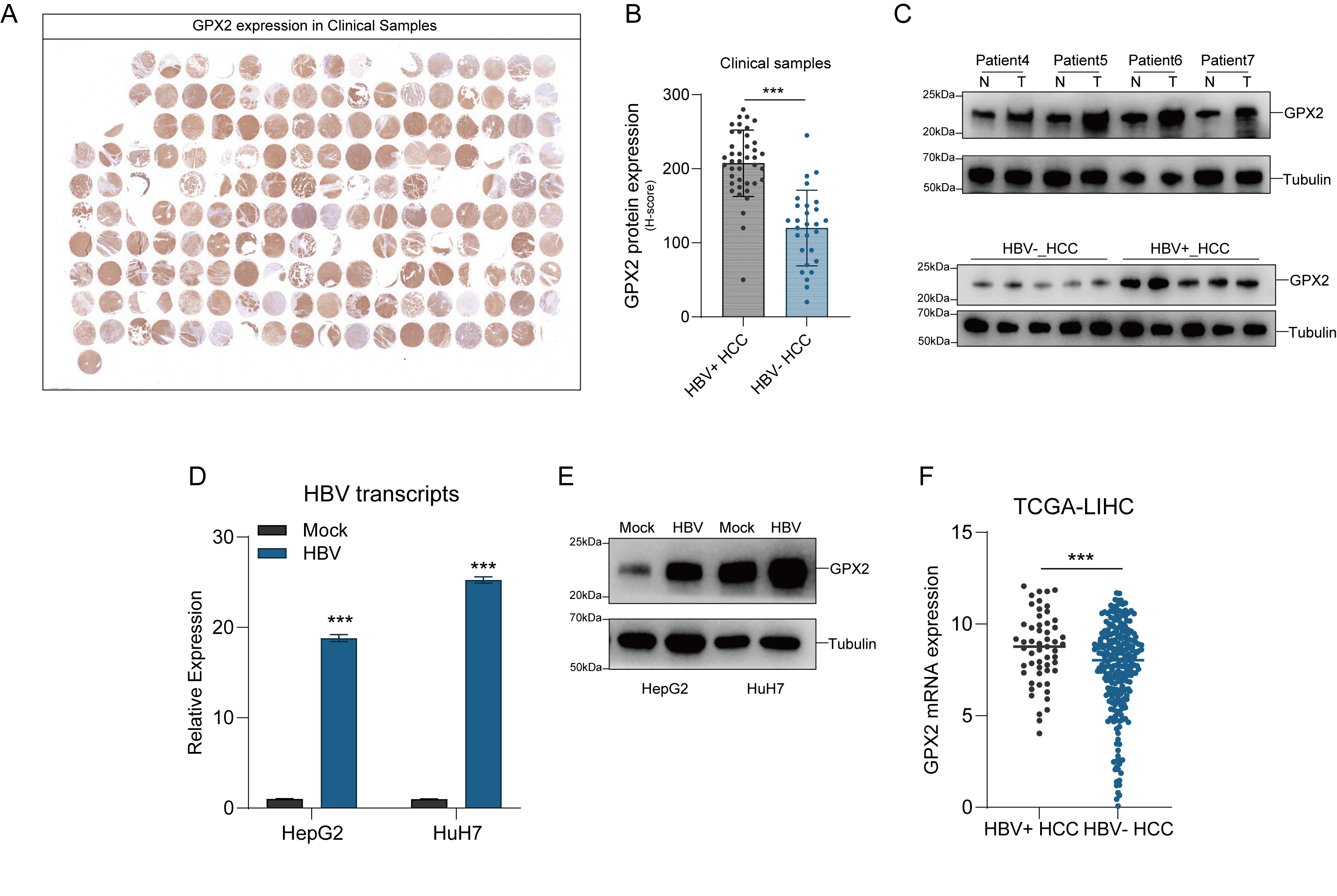

Supplement: Supplementary file 6 [file Image6.tif]
